# The impact of home- and community-based services on life satisfaction among older adults: evidence from marginal treatment effect framework

**DOI:** 10.3389/fpubh.2026.1852355

**Published:** 2026-07-06

**Authors:** Anqin Zhu, Zeyu Kong, Chunfu Chen

**Affiliations:** 1 School of Government, Nanjing University, Nanjing, China; 2 Department of Stomatology, Xuyi People’s Hospital, Kangda College of Nanjing Medical University, Huai’an, China

**Keywords:** health status, home- and community-based services, life satisfaction, marginal treatment effect framework, older adults, psychological resilience, social participation

## Abstract

**Background:**

In the context of rapid population aging, home- and community-based services (HCBS) have become an important intervention for improving the quality of life among older adults. However, the causal impact and the heterogeneous effects of HCBS utilization on life satisfaction remain insufficiently explored.

**Methods:**

Data from the 2023 China Longitudinal Aging Social Survey (CLASS) were analyzed. An instrumental variable (IV) approach and the marginal treatment effect (MTE) framework were employed to empirically examine the main effects, mechanisms, and heterogeneous effects of HCBS utilization on life satisfaction. Additionally, policy simulations were conducted to explore how to optimize the role of HCBS in improving life satisfaction among older adults.

**Results:**

HCBS utilization significantly enhances the life satisfaction of older adults. MTE estimates reveal a pattern of positive selection on gains, indicating that older adults who are more likely to use HCBS benefit more from it. Mechanism analysis shows that heterogeneous health returns to HCBS utilization are the key underlying driver for positive selection on gains in terms of life satisfaction. Policy simulations demonstrate that increasing HCBS utilization rates improves life satisfaction among new users, and expanding the coverage of community service facilities, pension insurance, and medical insurance indirectly promotes life satisfaction by increasing HCBS uptake.

**Conclusion:**

This study suggests that HCBS utilization improves life satisfaction among older adults, and this effect exhibits heterogeneity characterized by a positive selection on gains. These findings contribute to the empirical understanding of HCBS effectiveness and offer policy implications for targeted interventions to optimize the healthcare service system.

## 1 Introduction

Population aging has become a global trend. Worldwide, the proportion of individuals aged 65 and older is projected to rise from 10.21% in 2024 to 16.33% by 2050 (1). In China, the aging process is particularly pronounced due to the long-standing one-child policy. The number of people aged 65 and above in 2024 exceeded 208 million, accounting for 14.67% of the total population, and this proportion is expected to rise to 30.92% by 2050 (1). Aging poses considerable challenges to individual well-being. As people age, they are more likely to experience declining physical health, cognitive impairment, reduced economic security, and the loss of social roles, all of which negatively affect their quality of life and subjective well-being (2). Meanwhile, the concurrent trends of decreasing household size and increasing old-age dependency ratios have eroded families’ capacity to provide care, thereby increasing demand for institutional care services. However, these services are often expensive and have limited accessibility, which heightens unmet care needs among older adults and further exacerbates their vulnerability (3). Therefore, understanding the determinants of older adults’ quality of life and developing targeted policy interventions is essential for addressing the challenges of population aging.

Life satisfaction, defined as an individual’s self-assessment of overall quality of life and well-being, is widely recognized as a core component of mental and social well-being and is considered one of the key indicators of successful aging (4). Existing research has shown that higher life satisfaction fosters a more optimistic outlook on life, which facilitates the accumulation of positive emotional experiences across various life domains and, in turn, contributes to better physical and mental health, reduced healthcare utilization, and a lower risk of mortality (5, 6). As life expectancy increases, individuals are increasingly motivated to pursue higher levels of life satisfaction (7). To date, most studies have emphasized the influence of individual factors (e.g., demographic characteristics, socioeconomic status, health conditions, and psychosocial resources) and family-level factors (e.g., marital status, household size, number of children, and living arrangements) on life satisfaction among older adults (8). However, these factors do not fully account for variations in older adults’ life satisfaction. Some studies have suggested that intrinsic traits (e.g., genotype, physiological constitution, and personality) and external contexts (e.g., economic growth, institutional quality, and public service provision) also exert independent effects on life satisfaction (9). Therefore, further research is needed to investigate the determinants of life satisfaction among older adults.

Home- and community-based services (HCBS) are a form of socialized care that uses community facilities and resources to provide care and assistance to older adults (10). HCBS combines a wide range of services, including daily living support, emotional care, and medical services (10, 11). These services can meet the multiple needs of older adults and support aging in place, thereby supplementing family care and acting as an effective substitute for institutional care, ultimately enhancing mental and social well-being (11). According to the bio-psycho-social model of successful aging, life satisfaction in later life results from the process of goal pursuance and need satisfaction, which is shaped by both personal disposition and social-structural constraints (12). HCBS can activate various biological, psychological, and social pathways, thus enhancing older adults’ capacity to pursue goals and satisfy psychological needs, and thereby contributing to improved life satisfaction (2, 11, 13). Specifically, HCBS can provide timely and affordable medical treatment and preventive care to improve physical health, and offer emotional support to alleviate anxiety and depression, thereby contributing to higher life satisfaction (14). Moreover, HCBS can promote functional independence by offering assistance with daily living, thereby enabling older adults to navigate challenges and life transitions with greater confidence, which in turn strengthens psychological resilience and enhances life satisfaction (2). Additionally, HCBS can support aging in place and facilitate the maintenance of social networks, thereby increasing opportunities for social participation and further promoting life satisfaction (11, 15). As China experiences rapid population aging, the government has accelerated the expansion of HCBS in recent years. In 2016, the Ministry of Civil Affairs and the Ministry of Finance jointly issued the “Notice on Central Government Financial Support for Pilot Reforms of Home- and Community-Based Care Services for Older Adults,” officially launching the national HCBS reform. This policy framework supports the development of community service facilities, the training of professional care personnel, the standardization of service provision, and the integration of medical and eldercare services, thereby enabling older adults to receive high-quality, affordable, accessible, and diverse eldercare and medical services within home and community settings. Since then, pilot programs have expanded from an initial 26 sites to full coverage across all provinces by 2021. In this context, examining the impact of HCBS on life satisfaction among older adults is crucial for identifying problems in policy implementation and designing optimization strategies, thereby promoting the quality of life and overall well-being of the aging population.

Currently, a large number of empirical studies have examined the effects of HCBS. Because developed countries introduced HCBS earlier, the relevant literature has accumulated substantial evidence on the multidimensional benefits of HCBS in these contexts (16–18). Nevertheless, with the rapid development of the HCBS system in China, a growing body of literature focusing on China has begun to comprehensively evaluate the diverse impacts of HCBS within its unique institutional context. For example, some studies suggest that China’s HCBS system integrates multiple services, including daily care, healthcare, emotional support, and social and recreational activities, thereby meeting the diverse needs of older adults and, in turn, improving physical and mental health, enhancing psychological resilience, and promoting social participation (2, 6, 10, 16). Moreover, some studies argue that HCBS, as a form of formal care, can partially substitute for informal family care, thereby alleviating caregivers’ financial and time burdens, increasing caregivers’ labor supply, and improving intergenerational relationships (19–22). Furthermore, some studies suggest that HCBS helps to relax financial constraints related to healthcare and loosen older adults’ budget constraints, thereby increasing their total consumption expenditure, optimizing their consumption structure, and raising the share of leisure- and development-oriented consumption (23–25).

Moreover, because life satisfaction reflects individuals’ overall well-being across multiple domains of life (5), including health, economic status, intergenerational relations, and social interactions, this concept has attracted extensive attention in the literature. However, existing studies have not reached a consensus on how HCBS affects life satisfaction among older adults. One view is that HCBS can meet older adults’ daily living, medical, and social needs, enabling them to maintain independent living within familiar environments such as their homes and communities, thereby enhancing life satisfaction. This view has been supported by a growing body of recent empirical evidence. For instance, Jiang et al. (6), Liu and Yang (26), and Ma et al. (27) all employ multivariate linear regression models and find a statistically significant positive association between HCBS and older adults’ life satisfaction. Zhang et al. (28) and Jia et al. (29) use propensity score matching (PSM) to mitigate selection bias arising from observable characteristics and further confirm that HCBS significantly predicts higher life satisfaction among older adults. Moreover, Song and Li (2) and Sun (30), using longitudinal data and controlling for individual fixed effects to address time-invariant unobserved confounding, find that HCBS significantly improves older adults’ life satisfaction. In addition, Su et al. (11) and Lu and Chen (23) exploit the HCBS pilot program launched in China in 2016 as a quasi-experiment and, using a difference-in-differences (DID) design, demonstrate that the pilot policy significantly improves older adults’ life satisfaction. In contrast, another perspective highlights concerns such as inadequate service provision and mismatches between service supply and demand, which may result in inefficiencies or failures in meeting older adults’ needs and thereby limit the impact of HCBS on life satisfaction. This view is supported by a limited number of empirical studies. For example, Cheung et al. (7), using multivariate regression models, find that the utilization of specific types of community caring services is significantly negatively associated with older adults’ quality of life. Lu et al. (31), using longitudinal data, show that unmet service needs arising from mismatches between community service provision and older adults’ demands are significantly associated with lower baseline life satisfaction.

A plausible explanation for these mixed findings is that some studies do not adequately address the endogeneity of HCBS utilization, implying that their results should be interpreted as associations rather than causal effects (6, 7, 26, 27), which also reduces the comparability of findings across studies. Meanwhile, although some studies adopt HCBS pilot programs as quasi-experiments for causal inference, the treatment variable in such studies is typically defined at the macro level (23), yielding only intention-to-treat (ITT) estimates of the effect of HCBS pilots on older adults’ life satisfaction, which further limits their comparability with the broader literature that primarily focuses on micro-level measures of HCBS utilization. In addition, the mixed evidence regarding the effectiveness of HCBS utilization suggests potential heterogeneity in its impact on life satisfaction. Specifically, older adults’ sociodemographic characteristics, as well as the price, quality, and accessibility of HCBS, may all moderate life satisfaction gains from HCBS utilization. However, some studies are based on samples of older adults who are low-income, homeless, living alone, or suffering from chronic conditions (18, 32, 33), while others rely on data from a single province or from early stages of HCBS development in China (7, 10, 31, 34). Such differences in sample composition may imply that the reported findings primarily reflect the effects of HCBS on the life satisfaction of specific subgroups. Therefore, it is necessary to use nationally representative data and to fully account for potential endogeneity and treatment effect heterogeneity when further examining the impact of HCBS utilization on older adults’ life satisfaction.

Theoretically, the person-environment fit perspective provides an important lens for understanding heterogeneous effects of HCBS utilization on older adults’ life satisfaction. Specifically, this framework emphasizes that older adults’ well-being depends on the interaction between personal competence and environmental characteristics (18, 35, 36). As older adults experience age-related resource loss and functional declines, a supportive environment characterized by accessible community services becomes crucial for compensating for their vulnerabilities, enhancing residential and financial independence, facilitating social adaptation, and thereby maintaining and improving life satisfaction (35, 37, 38). From this theoretical perspective, the heterogeneous effects of HCBS utilization on older adults’ life satisfaction may arise from observable sociodemographic characteristics. Older adults who are socioeconomically disadvantaged due to gender, ethnicity, place of residence, social class, or family resource endowments are more likely to rely on, and thus potentially benefit more from, inclusive HCBS programs provided by the government. Meanwhile, unobserved psychological and cultural factors, such as stigma associated with reliance on non-family care or preferences for high-quality and specialized services, may act as latent constraints and make it difficult for some older adults to fully benefit from HCBS. Furthermore, unobserved supply-side factors (e.g., the price, accessibility, and quality of HCBS) may also contribute to heterogeneous effects on older adults’ life satisfaction. Lower service prices, together with greater accessibility and higher service quality, can stimulate utilization and enable older adults to obtain greater returns from HCBS. Overall, differences in both observable and unobservable characteristics jointly lead to heterogeneity in the effects of HCBS utilization on life satisfaction among older adults.

In addition, according to the bio-psycho-social model of successful aging (12), HCBS not only directly influences older adults’ life satisfaction but also generates a series of positive bio-psycho-social effects, including improvements in physical and mental health, strengthened psychological resilience, and increased social participation, thereby indirectly enhancing life satisfaction. However, from a person-environment fit perspective, these bio-psycho-social outcomes are likewise shaped by the fit between individuals’ competence and their surrounding environment (35, 36, 39). Therefore, as a critical form of environmental support, HCBS is unlikely to exert homogeneous effects on bio-psycho-social outcomes (18). Specifically, socioeconomically disadvantaged older adults often lack access to healthcare services, psychological support, and social participation. The inclusive services provided by HCBS are therefore more likely to compensate for their deficits in health, psychological resources, and social capital, thereby generating greater improvements in life satisfaction. Moreover, when HCBS programs are characterized by lower prices, greater accessibility, and higher service quality, they are more effective in reducing unmet health, psychological, and social needs among older adults, thereby yielding larger gains in life satisfaction. Such person–environment fit or misfit arising from both supply- and demand-side characteristics may consequently lead to heterogeneity in the effectiveness of HCBS in improving health status, enhancing psychological resilience, and facilitating social participation. Consistent with these theoretical expectations, existing empirical studies have shown that the effects of HCBS utilization on bio-psycho-social outcomes vary across older adults according to gender, age, educational level, economic status, urban–rural residence, and intergenerational living arrangements (40–44). Therefore, it is reasonable to expect that both observable and unobservable supply- and demand-side characteristics may further moderate the bio-psycho-social effects of HCBS utilization, thereby contributing to heterogeneity in its effects on older adults’ life satisfaction.

At present, several empirical studies based on nationally representative data have examined the heterogeneous effects of HCBS utilization on life satisfaction among older adults and have identified treatment effect heterogeneity across a range of dimensions, including demographic characteristics, socioeconomic status, family structure, and health conditions (11, 23, 26–30). However, differences in empirical design across studies, particularly the fact that endogeneity is not fully addressed in some studies, limit the comparability and credibility of the existing evidence. Nevertheless, a recent study employing a longitudinal design with fixed-effects models, which provides estimates with stronger causal interpretability, found that HCBS utilization generated greater improvements in life satisfaction among disadvantaged groups, including women, rural residents, low-income older adults, empty-nest older adults, and those with chronic conditions (30). These findings provide preliminary evidence supporting a pattern of positive selection in returns to HCBS utilization. However, existing heterogeneity analyses primarily rely on subgroup analyses or interaction terms, which can capture only heterogeneity driven by observable characteristics (i.e., observable heterogeneity) while failing to identify heterogeneity arising from unobservable factors (i.e., essential heterogeneity). Therefore, further research is needed to examine the causal and heterogeneous effects of HCBS utilization on life satisfaction among older adults, thereby providing empirical evidence to inform the optimization of service systems and enhance older adults’ psychological well-being.

**Figure 1:**
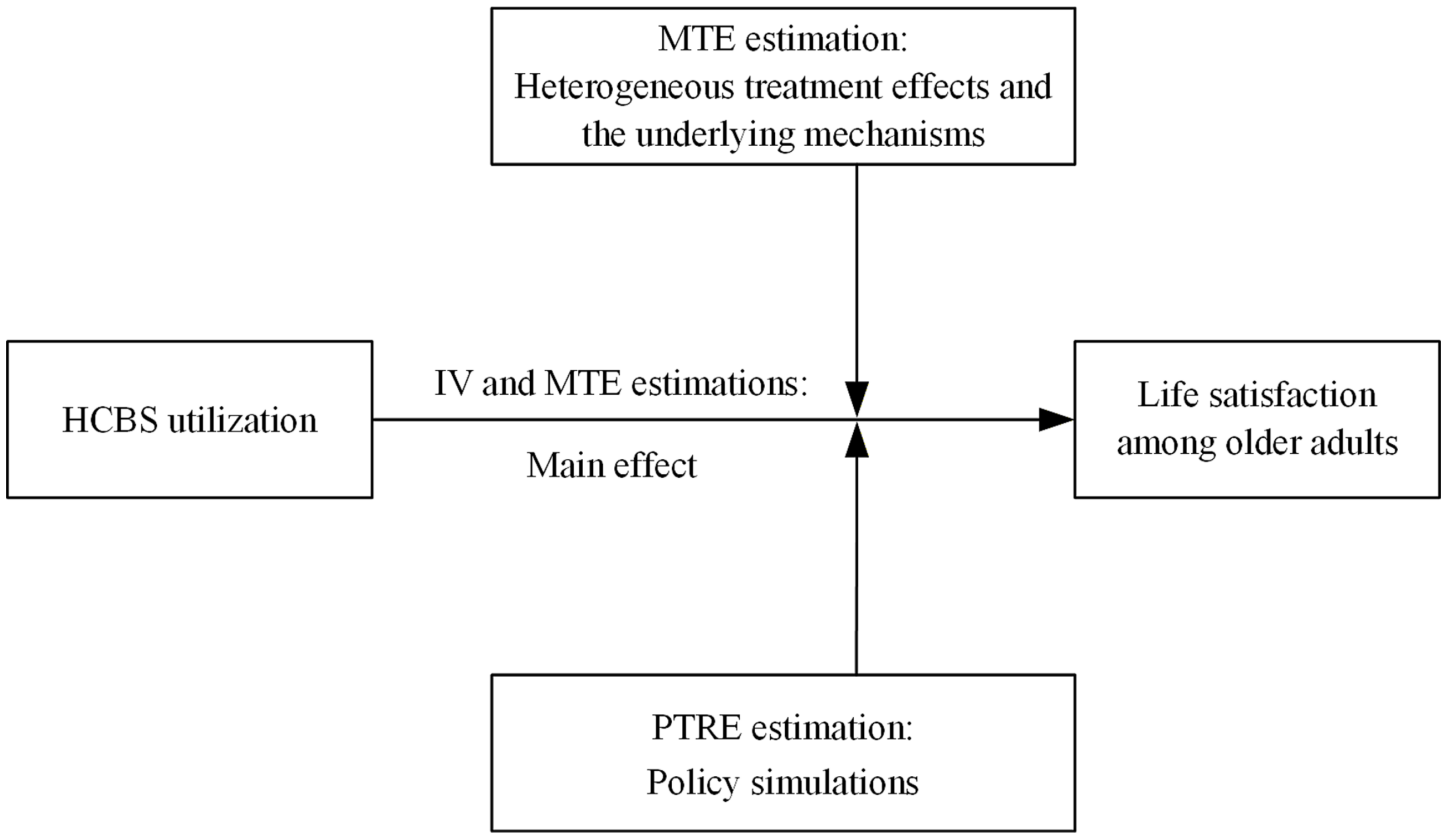
Analytical framework. The figure presents the different components of the current study. Specifically, IV estimation is used to provide preliminary evidence on the causal effect of HCBS utilization. MTE estimation is employed to extrapolate the treatment effects identified through the IV approach, thereby generating average treatment effect estimates with greater external validity. In addition, MTE estimation is used to uncover treatment effect heterogeneity in the impact of HCBS utilization on life satisfaction arising from both observable and unobservable characteristics, as well as the underlying mechanisms. Finally, PRTE estimation is used to simulate the expected effects of policy interventions aimed at increasing HCBS uptake.

To address the limitations of the existing literature, this study employs nationally representative data from the 2023 China Longitudinal Aging Social Survey (CLASS) and applies the marginal treatment effect (MTE) framework to empirically examine the impact of HCBS utilization on life satisfaction among older adults. Specifically, we first employ an instrumental variable (IV) approach to provide preliminary evidence on the main effect of HCBS utilization on life satisfaction and rigorously assess the validity of the IV. We then adopt the MTE framework to link heterogeneity in treatment effects to both observed and unobserved heterogeneity in the propensity to utilize HCBS, thereby offering a more comprehensive understanding of treatment effect heterogeneity. Moreover, the MTE framework enables us to extrapolate treatment effects from compliers identified in the IV framework to the full sample, thereby yielding more generalizable estimates of the average treatment effect. Third, we further investigate the sources of heterogeneity in the returns to HCBS utilization and uncover the roles of biological, psychological, and social pathways in shaping these differential returns. Finally, we conduct policy simulations by estimating policy-relevant treatment effects (PRTEs) to support the design of targeted interventions aimed at enhancing the effectiveness of HCBS and improving life satisfaction among older adults. Figure 1 illustrates our analytical framework and provides a clearer integration of the different components of the present study.

## 2 Methods

### 2.1 Data

The China Longitudinal Aging Social Survey (CLASS) is a national survey conducted by the National Survey Research Center at Renmin University of China. Given that the pilot program for HCBS had been extended to all provinces in China by 2021, this study employs the most recent publicly available wave of CLASS data from 2023 to ensure the timeliness and representativeness of the analysis. The 2023 CLASS dataset covers 28 provinces and includes a total of 11,670 respondents. It collects comprehensive information on respondents’ sociodemographic characteristics, family structure, social security participation, community service utilization, and life satisfaction, which are well suited for examining the research questions in this study. Following common practice in prior studies (6, 10, 28, 34), we restrict the sample to individuals aged 60 and above and handle missing values using listwise deletion, excluding observations with missing values in the dependent variable, independent variable, instrumental variable, or control variables. The final sample consists of 9,916 respondents. The missing rate is approximately 15%, which is similar to that reported in comparable studies conducted in China (6, 10). Moreover, in the robustness checks, we further address potential sample attrition bias by employing inverse probability attrition weighting (IPAW) and the extreme bounds approach used by Blattman et al. (45).

### 2.2 Variables

#### 2.2.1 Dependent variable

The dependent variable is life satisfaction, measured by the question: “Overall, are you satisfied with your current life?” Respondents answered using a five-point Likert scale, ranging from 1 (very dissatisfied) to 5 (very satisfied). The single-item measure of life satisfaction has been widely used among older adults in China and has demonstrated good reliability and validity (46).

#### 2.2.2 Independent variable

The independent variable is HCBS utilization. China’s HCBS system integrates a wide range of services, including daily care services, rehabilitation and nursing services, cultural and recreational activities, and psychological support, with the aim of meeting the diverse needs of older adults. For reasons of policy relevance, we follow the existing literature (20, 30) and construct the HCBS utilization measure using all 17 types of community services covered in the 2023 CLASS questionnaire. Specifically, the questionnaire asked respondents whether they had used any of the following 17 types of community services: home nursing, home medical visits, rehabilitation training, rental of rehabilitation assistive devices, free physical examinations, establishment of health records, health lectures, family doctor contracting services, home visits, older adult service hotlines, accompanied medical visits, assistance with daily shopping, legal aid, housekeeping services, meal delivery, day care, and psychological counseling. In addition, the MTE framework is applicable only to binary treatments, and previous studies have commonly operationalized HCBS utilization as a binary indicator (20, 30, 32, 33). To ensure the feasibility of the empirical strategy and to facilitate comparability with the existing literature, we likewise construct HCBS utilization as a binary variable. Specifically, the variable is coded as 1 if a respondent reported using at least one of these services, and 0 otherwise.

#### 2.2.3 Instrumental variable

Following the approach of existing literature (47, 48), this study uses community service facilities as the instrumental variable (IV). Specifically, if a respondent’s community has at least one of the following seven types of service facilities, including community activity rooms, fitness facilities, chess and card rooms, libraries, outdoor activity spaces, community canteens, and community care centers, the IV is coded as 1; otherwise, it is coded as 0.

There are two main reasons for using community service facilities as the IV. First, such facilities constitute the material foundation for the provision of HCBS and directly affect service accessibility, thereby satisfying the relevance condition. Moreover, it should be noted that community service centers for older adults in China generally follow an integrated and embedded development model, under which multiple functional spaces are combined within a single facility to provide daily care services, rehabilitation and nursing services, cultural and recreational activities, and psychological support, with the aim of meeting the diverse needs of older adults. Additionally, from the perspective of policy implementation, community service centers across provinces with different levels of economic development—such as Jiangsu in eastern China, Henan in central China, and Xinjiang in western China—typically include not only dining and caregiving spaces designed to meet older adults’ basic material needs, but also facilities such as libraries, chess and card rooms, and fitness rooms intended to satisfy their social and psychological needs (49–51). Therefore, the distinctive design and implementation of China’s HCBS system further strengthen the relevance of the IV. Second, as the community functions as the most basic administrative unit within China’s governance system, the construction of community service facilities is largely determined by higher-level governments and influenced by factors such as fiscal capacity, population size, and community area, rather than individual-level characteristics. Therefore, these facilities are unlikely to affect life satisfaction through channels other than HCBS utilization, meeting the exclusion restriction.

In addition, we further examine the validity of the IV through a series of empirical tests. Regarding the relevance condition, we directly assess its plausibility using weak identification tests and evaluate the robustness of the IV estimates to potential weak instrument problems through the Anderson-Rubin Wald test and limited information maximum likelihood (LIML) estimation. Regarding the exclusion restriction, we provide evidence supporting its plausibility using the variance-weighted Kolmogorov–Smirnov test, the intersection bounds approach, and placebo tests, and further assess the robustness of the IV estimates to relaxations of the exclusion restriction through two sensitivity analyses. Through these tests, we provide substantial evidence supporting the validity of the IV, thereby enhancing the credibility of the empirical results.

#### 2.2.4 Control variables

To reduce omitted variable bias, we control for a set of confounding variables based on previous research (52), including: gender (female = 0, male = 1), age, ethnicity (Han = 0, ethnic minority = 1), household registration (*hukou* in Chinese) type (agricultural household registration = 1, non-agricultural household registration = 0), marital status (single = 0, married = 1), political affiliation (non-member = 0, Chinese Communist Party member = 1), education (measured by years of schooling), household financial status (measured as the natural logarithm of annual per capita household consumption), pension insurance (non-participant = 0, participant = 1), medical insurance (non-participant = 0, participant = 1), number of houses, household size, number of children, and living arrangement (not living with children = 0, living with children = 1). To account for unobserved macro-level factors, province fixed effects are also included.

#### 2.2.5 Mechanism variables

To explain the potential mechanisms underlying the heterogeneous effects of HCBS on older adults’ life satisfaction, we draw on the bio-psycho-social model of successful aging (12) and, subject to data availability, select three groups of mechanism variables: (i) physical and mental health, including self-rated health (SRH), activities of daily living (ADL), instrumental activities of daily living (IADL), and depression; (ii) psychological resilience; and (iii) social participation.

Specifically, SRH is measured based on the question: “How would you rate your current physical health status?” Respondents answered on a five-point Likert scale ranging from 1 (very poor) to 5 (very good). ADL is measured using the Katz Scale, which includes six basic tasks such as eating, dressing, and bathing. The ADL score is calculated as the total number of activities that the respondent can perform independently. IADL is measured using the Lawton Scale, which includes eight tasks such as making phone calls, taking medication, and shopping. The IADL score is calculated as the total number of activities that the respondent can perform independently. Depression is measured using the nine-item version of the Center for Epidemiologic Studies Depression (CES-D) Scale. Respondents rated each item on a three-point Likert scale ranging from 1 (rarely or never) to 3 (often). The total score is used as the depression variable. Psychological resilience is measured using the eight-item scale, which assesses an individual’s capacity to accept changes in personal life and society, and has demonstrated good reliability and validity in the Chinese context (53). Respondents evaluated each statement on a five-point Likert scale ranging from 1 (strongly disagree) to 5 (strongly agree), and the average score is used as the psychological resilience variable. Social participation is measured by the average frequency of participation in six types of activities, including religious, educational, and recreational activities. Responses range from 0 (never) to 4 (almost daily).

### 2.3 Models

This study aims to examine the causal and heterogeneous effects of HCBS utilization on life satisfaction among older adults. We begin by considering the following ordinary least squares (OLS) model:


$$Y_{i}=a_{0}+a_{1}D_{i}+a_{2}X_{i}+\varepsilon_{i}\#$$ (1)

Where $Y_{i}$ denotes life satisfaction, $D_{i}$ indicates HCBS utilization, $X_{i}$ is a vector of control variables, and $\varepsilon_{i}$ is the error term. However, Equation (1) may suffer from endogeneity arising from unobservable omitted variables. On the one hand, previous studies have shown that household economic conditions play an important role in older adults’ decisions to utilize HCBS. Although this study controls for several variables reflecting household economic status, including education, household financial status, and number of houses, data limitations prevent the inclusion of other forms of household resource endowments, such as bank deposits, stocks, and social capital. These factors may also affect older adults’ life satisfaction, thereby giving rise to omitted variable bias. On the other hand, HCBS-related factors, such as service price and quality, as well as the broader policy environment, which includes local governments’ political attention to community-based care services and the intensity of fiscal investment in such services, may simultaneously influence both older adults’ willingness to use HCBS and their life satisfaction, further exacerbating the omitted variable problem.

To address the endogeneity, we employ an IV approach and estimate a two-stage least squares (2SLS) model as follows:


$$D_{i}=b_{0}+b_{1}IV_{i}+b_{2}X_{i}+\zeta_{i}$$ (2)

$$\begin{array}{c} Y_{i}=c_{0}+c_{1}\hat{D_{i}}+c_{2}X_{i}+\eta_{i}, \end{array}\;$$ (3)

Where $IV_{i}$ represents the community service facilities, and $\hat{D_{i}}$ is the fitted value from the Equation 2. When the IV satisfies the relevance and exclusion restrictions, the coefficient $c_{1}$ in Equation 3 identifies the causal effect of HCBS utilization on life satisfaction.

However, the IV approach typically estimates one overall effect, and thus fails to detect the treatment effect heterogeneity. Conventional methods for assessing heterogeneity (e.g., subgroup analysis or interaction terms) are limited to heterogeneity driven by observed characteristics and are insufficient when treatment effects are conditional on unobserved variables. Moreover, in the presence of treatment effect heterogeneity, the IV approach identifies only the local average treatment effect (LATE) for individuals whose treatment status is influenced by the instrument (i.e., the compliers), which may limit the external validity of the IV estimates.

In this study, we employ the marginal treatment effect (MTE) framework to address the issues mentioned above. Specifically, the MTE framework is based on the following generalized Roy model:


$$\begin{array}{c} Y_{1}=\beta_{1}X+U_{1} \end{array}$$ (4)

$$\begin{array}{c} Y_{0}=\beta_{0}X+U_{0} \end{array}$$ (5)

$$\begin{array}{c} D=1[P(Z)-V\geq 0] \end{array}$$ (6)

Where $Y_{1}$ and $Y_{0}$ represent the potential outcomes under treated and untreated states, respectively; X denotes observed control variables; $U_{1}$ and $U_{0}$ are unobserved variables; Z includes both control variables and instrumental variables; P(Z) is the propensity score, i.e., the probability of treatment given Z ; and V represents unobserved resistance to treatment. In this study, V primarily captures factors such as service costs, service quality, and expected benefits from using the services. The indicator function 1[∗] takes the value 1 if P(Z)−V≥0 , implying that the individual chooses to utilize HCBS, and 0 otherwise.

Assuming that V obeys a uniform distribution on the interval [0, 1], and letting $U_{D}$ denote the quantile of V , the MTE is defined as the treatment effect at $P(Z)=U_{D}$ . Combining Equations 4–6, the MTE is derived in Equation 7:


$$\begin{array}{c} \begin{array}{l} \mathrm{MTE}(x,u)=E(Y_{1}-Y_{0}|,X=x|,U_{D}=u)\\ =x(\beta_{1}-\beta_{0})+E(U_{1}-U_{0}|U_{D}=u) \end{array} \end{array}$$ (7)

Where $x(\beta_{1}-\beta_{0})$ captures the observable heterogeneity, while $E(U_{1}-U_{0}|U_{D}=u)$ represents the essential heterogeneity. Thus, the MTE framework allows for treatment effects to vary with both observed factors and unobserved resistance to treatment, providing a comprehensive distribution of treatment effects.

Given that the IV is binary, we adopt the separate approach proposed by Brinch et al. (54) to estimate the MTE. This approach involves two steps. First, the propensity score P(Z) is estimated using a Probit model. Second, the conditional expectations of $Y_{1}$ and $Y_{0}$ are estimated separately for treated and untreated groups See Equations 8, 9:


$$\begin{array}{c} E(Y_{1}|X=x,P(Z)=p,D=1)=x\beta_{1}+E(U_{1}|U_{D}\leq p) \end{array}$$ (8)

$$\begin{array}{c} E(Y_{0}|X=x,P(Z)=p,D=0)=x\beta_{0}+E(U_{0}|U_{D}>p) \end{array}\;$$ (9)

$$\text{Letting,}K_{1}(p)=E(U_{1}|U_{D}\leq p)\;\text{and}K_{0}(p)=E(U_{0}|U_{D}>p)\text{their derivatives with respect to }p$$

are:


$$\begin{array}{c} k_{1}(p)=K_{1}(p)+pK_{1}^{\prime }(p) \end{array}\;$$ (10)

$$\begin{array}{c} k_{0}(p)=K_{0}(p)-(1-p)K_{0}^{\prime }(p) \end{array}$$ (11)

Because the MTE framework assumes that unobserved resistance to treatment follows a uniform distribution on the interval [0, 1], Equations 10, 11 can be derived using the Leibniz integral rule and the product rule. Given the applied nature of the present study, a detailed derivation of Equations 10, 11 is beyond the scope of this paper; the full derivation can be found in Brinch et al. (54). Nevertheless, the economic intuition behind Equations 10, 11 is relatively straightforward. Specifically, the MTE captures the treatment effect for individuals who are at the margin of indifference between using and not using HCBS (i.e., $P(Z)=U_{D}$ ). In this context, $K_{1}(p)$ and $K_{0}(p)$ can be interpreted as describing how life satisfaction for HCBS users and non-users varies with unobserved resistance to treatment, respectively. Since these control functions essentially characterize potential outcomes under different treatment states, taking their derivatives yields the conditional expectations of $U_{1}$ and $U_{0}$ for individuals at the margin, denoted by $k_{1}(p)$ and $k_{0}(p)$ , respectively (54, 55). The difference between the two derivatives then gives the essential heterogeneity component of the MTE in Equation 7, thereby allowing us to characterize the variation of the MTE with respect to unobserved resistance to treatment. In addition, the propositions concerning the identification and estimation of the MTE through the derivatives of the two control functions, $K_{1}(p)$ and $K_{0}(p)$ , have been formally proven in Heckman et al. (56) and Carneiro et al. (57). Accordingly, we form the MTE as follows:


$$\begin{array}{c} \begin{array}{l} \mathrm{MTE}(x,u)=E(Y_{1}-Y_{0}|X=x,U_{D}=u)\\ =x(\beta_{1}-\beta_{0})+k_{1}(p)-k_{0}(p) \end{array} \end{array}$$ (12)

To estimate the MTE in Equation 12, we need to make assumptions on the function K(p) . Following existing literature (58, 59), we assume K(p) to be a quadratic polynomial in the propensity score. We also conduct robustness checks using alternative model specifications to further verify the reliability of the MTE estimation results.

Moreover, the MTE framework allows the average treatment effect (ATE), the average treatment effect on the treated (ATT), and the average treatment effect on the untreated (ATUT) to be expressed as weighted averages of the MTE:


$$\begin{array}{c} \begin{array}{l} \mathrm{ATE}=\frac{1}{N}\sum_{i=1}^{N}X_{i}(\beta_{1}-\beta_{0})+\frac{1}{100}\sum_{i=1}^{100}\\ \left((U_{1}-U_{0})|U_{D}=\frac{u}{100}\right) \end{array} \end{array}$$ (13)

$$\begin{array}{c} \begin{array}{l} \mathrm{ATT}=\frac{1}{N}\sum_{i=1}^{N}\frac{p_{i}}{\bar{p}}X_{i}(\beta_{1}-\beta_{0})+\sum_{i=1}^{100}\frac{P(p>\frac{u}{100})}{100\bar{p}}\\ \left((U_{1}-U_{0})|U_{D}=\frac{u}{100}\right) \end{array} \end{array}$$ (14)

$$\begin{array}{c} \begin{array}{l} \text{ATUT}=\frac{1}{N}\sum_{i=1}^{N}\frac{1-p_{i}}{1-\bar{p}}X_{i}(\beta_{1}-\beta_{0})+\sum_{i=1}^{100}\frac{P(p\leq \frac{u}{100})}{100\bar{p}}\\ \left((U_{1}-U_{0})|U_{D}=\frac{u}{100}\right) \end{array} \end{array}$$ (15)

The ATE in Equation 13 reflects the expected impact of HCBS utilization on life satisfaction across the entire sample. By recovering the ATE, the MTE framework overcomes the limitation of the IV approach, which identifies only the LATE, thereby improving the external validity of the estimated treatment effects. The ATT in Equation 14 and ATUT in Equation 15 capture the expected impacts of HCBS utilization on life satisfaction for the treated and untreated groups, respectively. While the ATT is informative for evaluating the effectiveness of existing policies, the ATUT provides insights into the potential benefits of expanding such policies to individuals who have not yet used the services. Meanwhile, these summary treatment effect parameters can all be expressed as weighted averages of the MTE, where the weighting functions are derived from the conditional distributions of both the observed propensity score and unobserved resistance to treatment in the full sample, the treated group, and the control group. The detailed derivation of the PRTE weighting function can be found in Heckman and Vytlacil (60). With respect to the interpretation of the weighting functions, the ATE assigns equal weights to the MTE across individuals. By contrast, the ATT assigns greater weight to individuals with higher observed propensity scores and lower unobserved resistance to treatment, whereas the ATUT places greater weight on individuals with lower observed propensity scores and higher unobserved resistance to treatment. Thus, comparing ATT and ATUT allows us to uncover patterns of both observable and essential heterogeneity. Specifically, if ATT > ATUT, it suggests that those more likely to use HCBS benefit more from it, indicating a pattern of positive selection on gains. Conversely, if ATT < ATUT, it implies that individuals less likely to use HCBS would derive greater benefits, reflecting a negative selection pattern on gains.

Additionally, the MTE can be used to compute the policy-relevant treatment effect (PRTE) for policy simulations:


$$\begin{array}{c} \text{PRTE}=\frac{1}{N}\sum_{i=1}^{N}\frac{p_{i}^{\prime }-p_{i}}{\bar{p^{\prime }}-\bar{p}}X_{i}(\beta_{1}-\beta_{0})+\\ \sum_{i=1}^{100}\frac{P(p^{\prime }>\frac{u}{100})-P(p>\frac{u}{100})}{100\bar{p}}((U_{1}-U_{0})|U_{D}=\frac{u}{100}) \end{array}$$ (16)

In Equation 16, Where $p_{i}$ and $p_{i}^{\prime }$ denote the individual propensity scores before and after the policy change, while $\bar{p}$ and $\bar{p^{\prime }}$ represent the corresponding average propensity scores. The weighting function of the PRTE characterizes which individuals are newly induced into the treatment group as a result of policy changes. By applying this weighting function to the MTE and taking a weighted average, we obtain the treatment effect of HCBS utilization for the newly treated population. The detailed derivation of the PRTE weighting function can be found in Carneiro et al. (57). Based on this weighting function, the PRTE captures the average return to HCBS utilization for newly treated individuals under an alternative policy scenario, thereby allowing us to evaluate the expected impact of interventions aimed at increasing HCBS uptake (or, equivalently, raising the propensity score).

## 3 Results

### 3.1 Sample characteristics

Table 1 presents the descriptive statistics. The characteristics of older adults are reported using means and standard deviations (SD) for continuous variables and percentages (%) for categorical variables. Differences in characteristics between HCBS users and non-users are examined using t-tests for continuous variables and chi-square tests for categorical variables.

**Table 1:** Descriptive statistics.

Variables	Full sample	HCBS users	Non-HCBS users	Differences
	Mean (SD)/%	Mean (SD)/%	Mean (SD)/%	*p*-value
Life satisfaction	3.812 (0.745)	3.853 (0.745)	3.775 (0.742)	< 0.01
HCBS utilization				
Non-HCBS users	53.35%			
HCBS users	46.65%			
Community service facilities				< 0.01
No facility available	19.12%	14.03%	23.57%	
At least one facility available	80.88%	85.97%	76.43%	
Gender				0.14
Female	48.08%	48.88%	47.39%	
Male	51.92%	51.12%	52.61%	
Age	71.282 (5.939)	71.582 (5.991)	71.020 (5.881)	< 0.01
Ethnicity				0.01
Han	92.62%	91.89%	93.25%	
Ethnic minority	7.38%	8.11%	6.75%	
Household registration type				< 0.01
Agricultural household registration	58.26%	54.24%	61.78%	
Non-agricultural household registration	41.74%	45.76%	38.22%	
Marital status				0.24
Single	16.65%	17.12%	16.24%	
Married	83.35%	82.88%	83.76%	
Political affiliation				< 0.01
Non-member	96.99%	96.48%	97.45%	
Chinese Communist Party member	3.01%	3.52%	2.55%	
Education	6.409 (3.947)	6.487 (4.162)	6.340 (3.747)	0.07
Household financial status	9.144 (0.825)	9.203 (0.839)	9.093 (0.808)	< 0.01
Pension insurance				< 0.01
Non-participant	13.17%	8.47%	17.28%	
Participant	86.83%	91.53%	82.72%	
Medical insurance				< 0.01
Non-participant	4.62%	2.12%	6.81%	
Participant	95.38%	97.88%	93.19%	
Number of houses	1.094 (0.382)	1.104 (0.392)	1.086 (0.373)	0.02
Household size	2.515 (1.228)	2.597 (1.285)	2.443 (1.171)	< 0.01
Number of children	2.117 (1.135)	2.069 (1.155)	2.159 (1.116)	< 0.01
Living arrangement				< 0.01
Not living with children	75.87%	73.41%	78.02%	
Living with children	24.13%	26.59%	21.98%	
SRH	3.549 (0.770)	3.511 (0.778)	3.583 (0.762)	< 0.01
ADL	5.816 (0.662)	5.754 (0.741)	5.870 (0.579)	< 0.01
IADL	7.657 (1.141)	7.560 (1.282)	7.742 (0.994)	< 0.01
Depression	15.569 (3.339)	15.635 (3.333)	15.514 (3.344)	0.08
Psychological resilience	3.051 (0.499)	3.060 (0.472)	3.043 (0.521)	0.10
Social participation	0.975 (0.608)	1.041 (0.644)	0.917 (0.568)	< 0.01

The results show that 48.08% of the sample are female, and the average age is 71.282 years (SD = 5.939). Approximately 46.65% of older adults have used HCBS. On average, HCBS users report higher life satisfaction than non-users (*p* < 0.01). Compared with non-users, HCBS users are more likely to reside in communities with at least one type of community service facility (*p* < 0.01), providing preliminary evidence in support of the relevance of the IV. In addition, HCBS users are more likely to be older (*p* < 0.01), belong to ethnic minorities (*p* < 0.01), hold non-agricultural household registration (*p* < 0.01), be members of the Chinese Communist Party (*p* < 0.01), have higher educational attainment (*p* = 0.07), have better household financial status (*p* < 0.01), participate in pension insurance (*p* < 0.01) and medical insurance (*p* < 0.01), own more houses (*p* = 0.02), have larger household sizes (*p* < 0.01), have fewer children (*p* < 0.01), and live with their children (*p* < 0.01). With respect to mechanism variables, HCBS users report lower SRH (*p* < 0.01), lower ADL (*p* < 0.01), and lower IADL (*p* < 0.01), as well as higher levels of depression (*p* = 0.08), psychological resilience (*p* = 0.10), and social participation (*p* < 0.01), compared with non-users.

However, given the systematic differences in characteristics between HCBS users and non-users, and the potential correlation of these characteristics with both the outcome and mechanism variables, endogeneity concerns may arise, thereby confounding the true effect of HCBS utilization on these variables. Moreover, the effects of HCBS utilization may be heterogeneous across different groups of older adults. Therefore, in the subsequent analysis, we first estimate OLS models to preliminarily explore the association between HCBS utilization and older adults’ life satisfaction after controlling for observable characteristics. We then employ the IV approach to mitigate potential endogeneity concerns. Finally, based on the MTE framework, we estimate treatment effect parameters with causal interpretation and external validity, thereby uncovering both the average and heterogeneous effects of HCBS utilization on life satisfaction.

### 3.2 OLS and IV estimations

Table 2 presents the OLS and IV estimation results. Column (1) reports the OLS estimates, showing that HCBS utilization is positively and significantly associated with life satisfaction among older adults (*β* = 0.076, *p* < 0.01).

**Table 2:** OLS and IV estimations.

Variables	OLS	2SLS: reduced-form	2SLS: first-stage	2SLS: second-stage	LIML	2SRI: ordered logit	2SRI: ordered probit
	(1)	(2)	(3)	(4)	(5)	(6)	(7)
HCBS utilization	0.076^***^ (0.017)			0.815^***^ (0.273)	0.815^***^ (0.273)	2.183^***^ (0.715)	1.120^***^ (0.395)
Community service facilities		0.066^***^ (0.021)	0.081^***^ (0.012)				
Gender	0.011 (0.015)	0.009 (0.015)	−0.021^**^ (0.009)	0.026 (0.017)	0.026 (0.017)	0.076^*^ (0.043)	0.038 (0.024)
Age	−0.004^***^ (0.001)	−0.004^***^ (0.001)	0.004^***^ (0.001)	−0.007^***^ (0.002)	−0.007^***^ (0.002)	−0.021^***^ (0.005)	−0.011^***^ (0.003)
Ethnicity	0.009 (0.032)	0.014 (0.032)	0.088^***^ (0.021)	−0.057 (0.042)	−0.057 (0.042)	−0.198^*^ (0.105)	−0.079 (0.059)
Household registration type	0.134^***^ (0.020)	0.131^***^ (0.020)	0.023^*^ (0.012)	0.112^***^ (0.023)	0.112^***^ (0.023)	0.351^***^ (0.059)	0.186^***^ (0.033)
Marital status	0.085^***^ (0.026)	0.086^***^ (0.026)	−0.013 (0.016)	0.097^***^ (0.029)	0.097^***^ (0.029)	0.245^***^ (0.071)	0.141^***^ (0.040)
Political affiliation	−0.012 (0.048)	−0.009 (0.048)	0.048^*^ (0.029)	−0.048 (0.054)	−0.048 (0.054)	−0.053 (0.134)	−0.048 (0.077)
Education	0.010^***^ (0.002)	0.010^***^ (0.002)	−0.000 (0.001)	0.010^***^ (0.003)	0.010^***^ (0.003)	0.024^***^ (0.007)	0.015^***^ (0.004)
Household financial status	−0.017 (0.012)	−0.020^*^ (0.012)	−0.001 (0.007)	−0.019 (0.013)	−0.019 (0.013)	−0.043 (0.034)	−0.032^*^ (0.019)
Pension insurance	0.061^**^ (0.028)	0.059^**^ (0.028)	0.043^***^ (0.016)	0.024 (0.033)	0.024 (0.033)	0.056 (0.085)	0.033 (0.046)
Medical insurance	0.059 (0.042)	0.075^*^ (0.042)	0.176^***^ (0.023)	−0.069 (0.066)	−0.069 (0.066)	−0.114 (0.166)	−0.071 (0.092)
Number of houses	0.060^***^ (0.022)	0.057^***^ (0.022)	0.000 (0.013)	0.057^**^ (0.025)	0.057^**^ (0.025)	0.217^***^ (0.061)	0.097^***^ (0.035)
Household size	−0.016 (0.013)	−0.016 (0.013)	0.008 (0.009)	−0.023 (0.014)	−0.023 (0.014)	−0.065^*^ (0.034)	−0.036^*^ (0.020)
Number of children	0.013 (0.008)	0.012 (0.008)	−0.015^***^ (0.005)	0.025^**^ (0.010)	0.025^**^ (0.010)	0.068^***^ (0.024)	0.035^***^ (0.014)
Living arrangement	0.005 (0.039)	0.007 (0.039)	−0.001 (0.025)	0.007 (0.043)	0.007 (0.043)	0.059 (0.103)	0.018 (0.059)
Constant	3.857^***^ (0.160)	3.842^***^ (0.161)	−0.115 (0.106)	4.017^***^ (0.193)	4.017^***^ (0.193)		
Province fixed effects	Yes	Yes	Yes	Yes	Yes	Yes	Yes
N	9,916	9,916	9,916	9,916	9,916	9,916	9,916
Kleibergen-Paap F-statistic	46.595						
Anderson-Rubin Wald test *p*-value	< 0.01						

^*^p < 0.1, ^**^p < 0.05, ^***^p < 0.01. Robust standard errors in parentheses.

Columns (2) to (4) report the 2SLS estimates. The reduced-form results indicate that community service facilities significantly increase life satisfaction among older adults (*β* = 0.066, *p* < 0.01). The first-stage regression results demonstrate that community service facilities significantly increase HCBS utilization (*β* = 0.081, *p* < 0.01). The Kleibergen-Paap F-statistic is 46.595, which exceeds the 10% critical value (16.38) from the Stock-Yogo weak identification test, thereby alleviating concerns about weak instruments. The second-stage regression results show that HCBS utilization significantly enhances life satisfaction among older adults (*β* = 0.815, *p* < 0.01). The magnitude of the effect is economically meaningful: HCBS users report life satisfaction levels that are, on average, 0.815 units higher than non-users, representing approximately 21.4% of the sample mean (3.812).

To further validate the relevance of the IV, we conducted the Anderson-Rubin Wald test, which is robust to weak instruments. The *p*-value is less than 0.01, leading us to reject the null hypothesis that the coefficient on HCBS utilization equals zero. In addition, we re-estimated the IV model using the LIML approach. Column (5) shows that the LIML estimates remain substantively similar to the 2SLS estimates, further confirming the robustness of our findings to potential weak instrument concerns.

In addition, given the ordinal nature of life satisfaction, we further estimate ordered Logit and ordered Probit models using a two-stage residual inclusion (2SRI) approach (61). Specifically, we first obtain the residuals from the first-stage IV regression and then include the estimated residuals as a control variable in the ordered models to estimate the effect of HCBS utilization on life satisfaction. Columns (6) and (7) show that the coefficient on HCBS utilization is positive and statistically significant, which is consistent with the qualitative findings from the linear IV model.

However, because the IV approach identifies the LATE, which may lack external validity when treatment effects are heterogeneous. This limitation will be addressed in the following section using the MTE method. It should be noted that the MTE framework is applicable only to linear models, and Ferrer-i-Carbonell and Frijters (62) show that treating life satisfaction as a cardinal rather than an ordinal variable does not substantially affect estimation results. Therefore, we continue to rely on linear specifications in the MTE estimation.

### 3.3 IV validity tests

The validity of the IV relies on the relevance and exclusion restrictions. While the relevance condition is well supported, we further assess the exclusion restriction to validate the instrument.

First, canonical overidentification tests are applicable only in settings where the number of instruments exceeds the number of endogenous regressors and under the assumption of homogeneous treatment effects. In contrast, in our setting, the number of instruments equals the number of endogenous regressors, and we allow for the presence of heterogeneous treatment effects. Therefore, overidentification tests are not suitable for assessing IV validity in our context. To address this limitation, Kitagawa (63) proposes a variance-weighted Kolmogorov–Smirnov test to formally evaluate the exclusion restriction. A key parameter determining the weights is the trimming constant *ξ*. Following the existing literature (63), we set ξ to 0.07, 0.3, and 1, respectively, and compute the corresponding bootstrap *p*-values. If the p-value is not statistically significant, we fail to reject the null hypothesis that the IV is valid. In addition, Mourifié and Wan (64) show that the testable implications of IV validity can be characterized as a set of conditional moment inequalities, which can be tested using the intersection bounds approach proposed by Chernozhukov et al. (65). If the lower intersection bound is below zero, we fail to reject the null hypothesis that the IV is valid. As shown in Supplementary Table 1, the *p*-values from the variance-weighted Kolmogorov–Smirnov tests exceed 0.1 across all values of *ξ*. Supplementary Table 2 indicates that the lower bounds are negative at all significance levels. Taken together, these findings provide evidence supporting the validity of our IV.

Second, we follow the placebo test proposed by Barron et al. (66) to further examine IV validity. Specifically, while preserving the overall distribution of the IV, we randomly assign placebo IV values across individuals and re-estimate the 2SLS specification. If the resulting 2SLS estimates remain statistically significant, this would cast doubt on the IV’s validity. In line with the procedure outlined by Young (67), we conduct 2000 replications to account for sampling variation. Supplementary Figure 1 displays the histogram of the 2000 placebo t-values. The results indicate that none of the absolute values of the placebo t-values exceed 1.64, suggesting that the estimated effects of HCBS utilization are not statistically significant across all placebo samples and thus supporting the validity of the IV.

Finally, we conduct two sensitivity analyses to examine the robustness of the 2SLS estimates when the strict exclusion restriction is relaxed. The first is the union of confidence intervals (UCI) method proposed by Conley et al. (68). This approach relaxes the exclusion restriction by allowing for a potential direct effect of the IV on the outcome, with coefficient gamma. Following the existing literature (69), we assume that gamma lies within the range of ±0.25 β_RF_, where β_RF_ denotes the coefficient on community service facilities from the reduced-form regression in Column (2) of Table 2. Supplementary Figure 2 plots the UCI of the HCBS coefficient under this setting. The results show that, across all values of gamma, the estimated impact of HCBS utilization on life satisfaction remains significantly positive, reinforcing the qualitative findings from the IV estimation.

The second approach follows Cinelli and Hazlett (70), which allows for the presence of an unobserved omitted variable that is correlated with both the outcome and the IV, thereby capturing potential violations of the exclusion restriction. This framework assesses how strong such an omitted variable would need to be in order to reduce the coefficient of HCBS utilization in the 2SLS model to zero. Since the 2SLS estimator is essentially the ratio of the reduced-form estimate to the first-stage estimate, testing how strong an omitted variable must be to drive the reduced-form coefficient β_RF_ to zero is equivalent to testing how strong it must be to reduce the 2SLS coefficient β_2SLS_ to zero. To enhance interpretability, following existing practice in the literature (71), we use key observed covariates as benchmarks to set plausible bounds on the strength of unobserved confounders. Specifically, we use age as the benchmark variable, as it is the only covariate in Table 2 that is statistically significantly associated with both the treatment and the outcome at the 1% level, making it a plausible proxy for an omitted variable. Following Cinelli and Hazlett (72), we further assume that the maximum explanatory power of the unobserved confounder is at most three times that of age. Under this assumption, the reduced-form coefficient β_RF_ is estimated at 0.065 and is statistically different from zero (SE = 0.020, *p* < 0.01). This result indicates that even an unobserved confounder as strong as three times age would not be sufficient to reduce β_RF_ (and, equivalently, β_2SLS_) to zero, thereby further confirming the robustness of the baseline findings to violations of the exclusion restriction.

Although we conducted various tests to provide evidence for the validity of the IV and to show that unobservable confounding factors are unlikely to drive our results, we acknowledge this choice of IV as one limitation of our identification strategy and recognize that there is still room to improve the rigor of the evidence.

### 3.4 MTE estimation

Table 3 presents the MTE estimation results. Column (1) reports the Probit estimates, showing that community service facilities significantly increase HCBS utilization (*β* = 0.247, χ^2^ = 45.54, *p* < 0.01), further confirming the relevance of the IV. Regarding the control variables, HCBS utilization is significantly positively associated with age (*β* = 0.013, *p* < 0.01), ethnicity (*β* = 0.245, *p* < 0.01), household registration type (*β* = 0.067, *p* < 0.1), political affiliation (*β* = 0.142, *p* < 0.1), pension insurance (*β* = 0.136, *p* < 0.01), and medical insurance (*β* = 0.593, *p* < 0.01), and significantly negatively associated with gender (*β* = −0.065, *p* < 0.05) and number of children (*β* = −0.045, *p* < 0.01).

**Table 3:** MTE estimation.

Variables	Probit model	MTE: β_0_	MTE: β_1_-β_0_
	(1)	(2)	(3)
Community service facilities	0.247^***^ (0.037)		
Gender	−0.065^**^ (0.028)	0.069^***^ (0.021)	−0.098^***^ (0.031)
Age	0.013^***^ (0.003)	−0.010^***^ (0.002)	0.007^**^ (0.004)
Ethnicity	0.245^***^ (0.058)	−0.209^***^ (0.049)	0.309^***^ (0.078)
Household registration type	0.067^*^ (0.036)	0.106^***^ (0.028)	0.020 (0.042)
Marital status	−0.039 (0.046)	0.069^*^ (0.037)	0.045 (0.053)
Political affiliation	0.142^*^ (0.083)	−0.015 (0.072)	−0.022 (0.098)
Education	−0.000 (0.004)	0.007^**^ (0.003)	0.006 (0.005)
Household financial status	−0.005 (0.022)	0.010 (0.016)	−0.071^***^ (0.025)
Pension insurance	0.136^***^ (0.048)	−0.044 (0.038)	0.181^***^ (0.064)
Medical insurance	0.593^***^ (0.084)	−0.209^***^ (0.071)	0.396^***^ (0.130)
Number of houses	0.002 (0.039)	0.097^***^ (0.030)	−0.061 (0.043)
Household size	0.022 (0.025)	0.003 (0.017)	−0.046^*^ (0.026)
Number of children	−0.045^***^ (0.014)	0.015 (0.012)	0.014 (0.018)
Living arrangement	0.002 (0.071)	−0.033 (0.051)	0.095 (0.078)
Constant	−1.900^***^ (0.316)	3.848^***^ (0.244)	0.103 (0.542)
Province fixed effects	Yes	Yes	
N	9,916	9,916	
ATE	0.536^**^ (0.256)		
ATT	1.414^***^ (0.315)		
ATUT	−0.232 (0.390)		
χ^2^ for test of the IV	45.54^***^		
Observable heterogeneity	<0.01		
Essential heterogeneity	<0.01		

*p < 0.1, **p < 0.05, ***p < 0.01. Robust standard errors in parentheses.

Column (2) reports *β*_0_ from the MTE specification, capturing the association between control variables and life satisfaction among non-HCBS users. The results indicate that life satisfaction is significantly positively associated with gender (*β* = 0.069, *p* < 0.01), household registration type (*β* = 0.106, *p* < 0.01), marital status (*β* = 0.069, *p* < 0.1), education (*β* = 0.007, *p* < 0.05), and number of houses owned (*β* = 0.097, *p* < 0.01), and significantly negatively associated with age (*β* = −0.010, *p* < 0.01), ethnicity (*β* = −0.209, *p* < 0.01), and medical insurance (*β* = −0.209, *p* < 0.01).

Column (3) presents β_1_-β_0_ from the MTE specification, capturing the heterogeneity in treatment effects driven by control variables. The results show that the returns to HCBS utilization are significantly positively associated with age (*β* = 0.007, *p* < 0.05), ethnicity (*β* = 0.309, *p* < 0.01), pension insurance (*β* = 0.181, *p* < 0.01), and medical insurance (*β* = 0.396, *p* < 0.01), while significantly negatively associated with gender (*β* = −0.098, *p* < 0.01), household financial status (*β* = −0.071, *p* < 0.01), and household size (*β* = −0.046, *p* < 0.1). The observable heterogeneity column reports the *p*-values from joint significance tests of the β_1_-β_0_ coefficients across all observed characteristics. The statistically significant p-values indicate that the effect of HCBS utilization on life satisfaction varies with observable characteristics. Notably, the coefficients for gender, age, ethnicity, pension insurance, and medical insurance are statistically significant and consistent in sign across Columns (1) and (3). This suggests that these variables not only increase the likelihood of using HCBS but also enhance its benefits, indicating a positive selection on gains based on observed characteristics.

**Figure 2:**
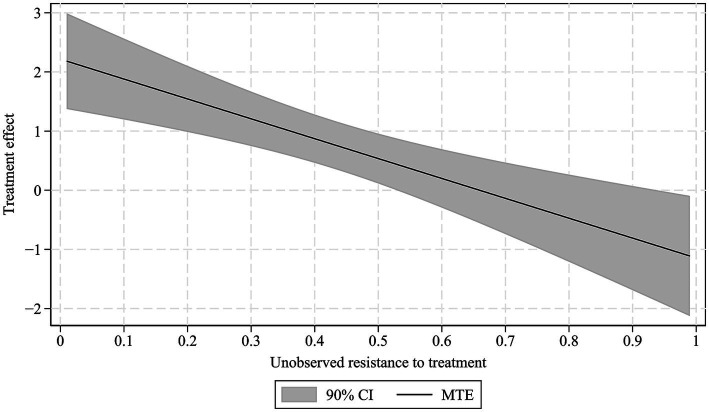
MTE curve. The figure plots the relationship between the MTE and unobserved resistance to treatment, including 90% confidence intervals for the MTE. The results show that, as unobserved resistance to treatment increases, the treatment effect gradually declines, indicating that the effect of HCBS utilization on older adults’ life satisfaction decreases as resistance increases.

Figure 2 illustrates the MTE curve, capturing heterogeneity in treatment effects driven by unobserved resistance to treatment. The results indicate that as the resistance increases, the treatment effect declines. In addition, the essential heterogeneity column reports the *p*-values from tests of whether the slope of the MTE curve equals zero. The statistically significant p-values indicate that the slope of the MTE curve differs significantly from zero, suggesting that the effect of HCBS utilization on life satisfaction also varies with unobserved characteristics. Taken together, these results suggest that older adults with unobserved characteristics that make them more likely to use HCBS benefit more from the service, demonstrating positive selection on gains based on unobserved characteristics.

Finally, summary treatment effect parameters are computed as weighted averages of the MTE. The ATE is 0.536 (*p* < 0.05), indicating that HCBS utilization significantly improves life satisfaction in the entire sample, amounting to approximately 14.1% of the dependent variable’s mean (3.812). This result supports the qualitative conclusions drawn from the OLS and IV estimations and lies quantitatively between the two. The ATT is 1.414 (*p* < 0.01), showing that for HCBS users, using the service increases life satisfaction by 1.414 units, approximately 37.0% of the dependent variable’s mean (3.812). The ATUT is −0.232 (*p* > 0.1), indicating that for non-users, HCBS does not significantly enhance life satisfaction. Since ATT assigns greater weights to individuals with higher observed propensity scores and lower unobserved resistance, whereas the ATUT places greater weights on those with lower observed propensity scores and higher unobserved resistance, the result that ATT > ATUT indicates that returns to HCBS utilization exhibit a pattern of positive selection on gains based on both observed and unobserved characteristics.

### 3.5 Robustness checks

Since the MTE framework not only yields estimates of average treatment effects with stronger causal interpretation and external validity, but also reveals heterogeneous treatment effects driven by both observable and unobservable characteristics, we conduct a series of robustness checks on the MTE results to further validate the credibility of the baseline findings.

#### 3.5.1 Addressing attrition bias

To address potential attrition bias arising from missing observations in the baseline analysis, we employ two complementary approaches: the inverse probability of attrition weighting (IPAW) method and the extreme bounds approach. To implement the IPAW procedure, we first examine the distribution of missing data and find that missing values in the main analytical sample are concentrated in two variables: life satisfaction and household financial status. Specifically, there are 58 missing observations for life satisfaction, accounting for approximately 0.5% of the full sample, and 1,688 missing observations for household financial status, accounting for approximately 14.5%. In contrast, all other variables used in the main analysis—including the independent variable, all control variables except household financial status, and province fixed effects—have no missing values. Based on this pattern of missing data, we construct weights for each observation used in the IPAW analysis. Specifically, we first define a binary indicator for whether an observation has missing values (missing = 1; non-missing = 0) and estimate a Logit model in which this indicator is regressed on all variables without missing values (including the independent variable, control variables excluding household financial status, and province fixed effects). We then obtain the predicted probability of attrition. Subsequently, we compute the probability of non-attrition as one minus the predicted probability of attrition and use the inverse of the probability of non-attrition as the weight for each observation. Finally, we apply these weights in the weighted MTE regression, thereby ensuring that the estimates are more representative of the full sample.

For the extreme bounds approach, following Blattman et al. (45), we impute missing values of the dependent variable under two extreme scenarios to compute bounds for the treatment effect. In the first scenario, for treated observations with missing values of the dependent variable, we assign the maximum value of 5, while for control observations with missing values, we assign the minimum value of 1. This procedure maximizes the estimated treatment effect among observations with missing outcomes and yields the upper bound estimates. In the second scenario, we reverse this procedure: for treated observations with missing values, we assign the minimum value of 1, while for control observations with missing values, we assign the maximum value of 5, thereby minimizing the estimated treatment effect among missing observations and yielding the lower bound estimates.

The results from both approaches are reported in Panel (A) of Supplementary Table 3. The ATE and ATT estimates are both positive and statistically significant. The ATUT estimate is statistically insignificant, and both observable and essential heterogeneity tests yield *p*-values below 0.01, thereby supporting the validity of the baseline MTE estimates.

It is worth noting that, due to the endogeneity concerns associated with OLS and the limited external validity of the LATE identified by the IV approach, we primarily rely on the MTE-based ATE estimates to capture the main effects of HCBS utilization on older adults’ life satisfaction and therefore do not separately report the robustness check results for OLS and 2SLS models in the table. Nevertheless, we implement the above approaches to further address potential attrition bias in both OLS and 2SLS estimates. The OLS estimates obtained under different methods are positive and statistically significant (IPAW: *β* = 0.094, SE = 0.020, *p* < 0.01; upper bound: *β* = 0.094, SE = 0.017, *p* < 0.01; lower bound: *β* = 0.056, SE = 0.017, *p* < 0.01). The corresponding 2SLS estimates are also robust (IPAW: *β* = 0.993, SE = 0.261, *p* < 0.01; upper bound: *β* = 0.838, SE = 0.279, *p* < 0.01; lower bound: *β* = 0.826, SE = 0.280, *p* < 0.01). These results further confirm the robustness of the baseline findings from both OLS and IV estimations.

Overall, after accounting for potential attrition bias, our main conclusions remain qualitatively unchanged. Moreover, from a quantitative perspective, the treatment effect parameters obtained from the MTE framework, which possess both causal interpretation and external validity, vary only modestly across specifications, suggesting that the results are not sensitive to missing data and are therefore highly robust.

#### 3.5.2 Assessing the functional form of the MTE curve

The credibility of MTE estimates depends on the plausibility of functional form assumptions. Accordingly, we examine the functional form of the MTE curve by comparing the LATE estimates obtained from the 2SLS specification and the MTE-weighted approach, following the method proposed by Cornelissen et al. (73). This method suggests that although the MTE curves derived under different functional form assumptions may yield different weighted LATEs, the LATE weights and the 2SLS estimator remain invariant to these assumptions. Therefore, a large discrepancy between the MTE-weighted LATE and the 2SLS LATE may indicate potential misspecification in the functional form of the MTE curve. Supplementary Figure 3 presents the LATE weights and estimates. The results indicate that the LATE estimator places greater weight on older adults with low to moderate resistance to HCBS utilization. Applying these weights to the MTE of compliers yields a weighted LATE of 0.799 (SE = 0.245, *p* < 0.01), which is close to the 2SLS estimate of 0.815 (*p* < 0.01), suggesting that the functional form of the MTE curve is unlikely to be misspecified.

#### 3.5.3 Alternative model specifications

To further rule out potential model misspecification bias, we assess the sensitivity of the baseline findings by considering alternative MTE specifications, including: (i) estimating the propensity score using both a Logit model and a linear probability model (LPM); (ii) specifying the K(p) function as a cubic polynomial of the propensity score; (iii) assuming a joint normal distribution of (U1, U0, V); and (iv) adopting a semiparametric estimation approach. Panel (B) of Supplementary Table 3 presents the MTE estimates under these alternative specifications. The ATE estimates are positive and statistically significant, indicating that HCBS utilization overall contributes to improved life satisfaction among older adults. The ATT estimates are significantly positive, while the ATUT estimates are negative but statistically insignificant, suggesting that older adults with a higher propensity to use HCBS benefit more from the services. Additionally, both the *p*-values for observable and essential heterogeneity are statistically significant, indicating that the positive selection pattern in returns to HCBS utilization is driven by both observable characteristics and unobserved resistance to treatment. Taken together, these results support the robustness of our baseline findings.

#### 3.5.4 Alternative operationalizations of the independent variable

The baseline analysis operationalizes HCBS utilization using a binary indicator, which may not fully capture the multidimensional nature of this concept. Accordingly, we consider two alternative operationalizations of the independent variable, including the category of HCBS utilization and the number of services utilized, to address potential measurement error. On the one hand, China’s HCBS system primarily consists of two components: medical services and eldercare services (20, 22, 30). The 2023 CLASS questionnaire asked respondents about their utilization of 8 types of medical services (including home nursing, home medical visits, rehabilitation training, rental of rehabilitation assistive devices, free physical examinations, establishment of health records, health lectures, and family doctor contracting services) and 9 types of eldercare services (including home visits, older adult service hotlines, accompanied medical visits, assistance with daily shopping, legal aid, housekeeping services, meal delivery, day care, and psychological counseling). We constructed binary indicators for each category of services, coded as 1 (used at least one service) and 0 (not used), and the utilization rates for medical services and eldercare services were 44.0 and 10.1%, respectively. We then estimated the MTE specifications using medical service utilization and eldercare service utilization, respectively, as the independent variable. It should be noted that the relatively low utilization rate of eldercare services results in limited common support in the propensity scores between the treatment and control groups. Consequently, treatment effects estimated using the parametric MTE model would rely heavily on extrapolation and thus lack credibility. Following the existing literature (74), we therefore employ a fully semiparametric MTE model to estimate treatment effects with greater internal validity for eldercare service utilization. The results are reported in Panel (C) of Supplementary Table 3. The low utilization rate of eldercare services reduces the weight of treated observations and leads to a statistically insignificant ATE. In contrast, the ATE for medical service utilization remains significantly positive. These findings suggest that medical services may be the primary driver of the positive and statistically significant average effects of HCBS utilization on older adults’ life satisfaction. Moreover, by controlling for province fixed effects in cross-sectional data, we alleviate concerns about confounding from other concurrent healthcare policies implemented at the macro level. In addition, by employing the MTE framework to obtain treatment effect parameters with both causal interpretability and external validity, we further address potential confounding from unobserved overlapping policy interventions. Consequently, our approach yields more credible estimates of the ATE. Nevertheless, the effects of HCBS utilization across different categories consistently exhibit a pattern of positive selection on gains. Overall, these findings provide support for the baseline conclusions.

On the other hand, given the methodological challenges associated with estimating the MTE with a multivalued treatment, and the fact that 85% of older adults in our sample used no more than two types of HCBS, we follow the approach of Cornelissen et al. (58) and categorize the treatment variable into three groups: no use, use of one type, and use of two or more types of HCBS. We then estimate two transition-specific MTE curves (Supplementary Figure 4) and the corresponding ATEs (Supplementary Table 4) using a generalized ordered choice Roy model. The results indicate that the MTE curve for the transition from no HCBS use to one type of HCBS is downward sloping, with a significantly negative slope and an associated ATE of 0.535 (*p* < 0.01). However, the MTE curve for the transition from one to two or more types of HCBS is relatively flat, and neither the slope nor the associated ATE is statistically significant. These findings suggest that the pattern of positive selection on gains is primarily concentrated along the extensive margin (i.e., no HCBS utilization vs. any HCBS utilization), rather than along the intensive margin (i.e., some HCBS utilization vs. more HCBS utilization), thereby supporting the validity of our baseline MTE specification, which relies on a binary treatment indicating whether individuals use HCBS or not.

#### 3.5.5 Alternative instrumental variables

The baseline analysis constructs the IV based on whether a respondent’s community contains any of the seven types of service facilities. However, substantial heterogeneity may exist across these facilities. Accordingly, we construct alternative IVs by classifying the service facilities into more precise categories and re-estimating the MTE specifications. Specifically, to minimize subjectivity in the classification process, we adopt a data-driven approach and conduct an exploratory factor analysis of the seven types of facilities, extracting two factors with eigenvalues greater than one. Community activity rooms, fitness facilities, chess and card rooms, libraries, and outdoor activity spaces exhibit factor loadings of 0.523, 0.637, 0.629, 0.651, and 0.571, respectively, on the first factor, all exceeding 0.5. In contrast, their loadings on the second factor are 0.287, 0.213, 0.325, 0.212, and −0.174, respectively, all below 0.5. By comparison, community canteens and community care centers exhibit loadings of 0.167 and 0.076, respectively, on the first factor, both below 0.5, while their loadings on the second factor are 0.874 and 0.886, respectively, both exceeding 0.5. Based on these results, we classify the first five types of facilities as “social and recreational facilities” and the latter two as “care and support facilities.”

Subsequently, we construct two alternative IVs based on the availability of these two categories of facilities within respondents’ communities and re-estimate the MTE specifications. Given that these two categories of facilities may affect HCBS utilization differently across population groups, we include both IVs in the first-stage regression to estimate the propensity scores, thereby expanding the common support region and enhancing the external validity of the MTE estimates. Panel (D) of Supplementary Table 3 shows that, after classifying community service facilities more precisely, the ATE and ATT remain positive and statistically significant, whereas the ATUT remains statistically insignificant. Moreover, the *p*-values for observable heterogeneity and essential heterogeneity remain statistically significant, further supporting the baseline findings.

#### 3.5.6 Changing the level of fixed effects

The baseline regressions account for unobserved macro-level characteristics by including province fixed effects. However, the provision and utilization of HCBS may also be influenced by more fine-grained regional characteristics, such as those at the city or community level, making it necessary to control for fixed effects at a more detailed level. Since the CLASS 2023 data do not publicly release city identifiers, this study further addresses the confounding influence of unobserved macro-level characteristics by controlling for community fixed effects. In addition, it should be noted that, because CLASS is a longitudinal panel survey, we also attempted to identify city-level information for our sample by linking respondents to earlier survey waves. However, longitudinal surveys involving older adults generally experience relatively high attrition rates. Moreover, the COVID-19 pandemic between 2020 and 2023 further intensified respondent attrition in the earlier CLASS waves. Specifically, in the current study, we matched respondents across survey waves using their personal identifiers and found that 3,803 observations (approximately 38.35% of our analytical sample) still lacked city-level information. More importantly, compared with the unmatched sample, the successfully matched sample differed significantly in both HCBS utilization and several individual characteristics. Therefore, conducting the empirical analysis based solely on the successfully matched sample would introduce substantial attrition bias and weaken the external validity and generalizability of the estimates. Accordingly, this study relies only on the community identifiers available in the CLASS 2023 data to conduct the robustness checks.

Panel (E) of Supplementary Table 3 shows that, after replacing province fixed effects with community fixed effects, the estimated ATE and ATT remain positive and statistically significant, whereas the ATUT remains statistically insignificant. In addition, the *p*-values for both observable and essential heterogeneity remain statistically significant. These findings further corroborate the baseline results.

Nevertheless, it should be noted that the first-stage regression in the MTE estimation employs a Probit model to estimate propensity scores. In nonlinear models such as Probit, controlling for more fine-grained community fixed effects results in a substantial number of observations (*N* = 517, accounting for approximately 5% of the baseline sample) being automatically dropped due to “perfect prediction” (i.e., all individuals within a given community either used or did not use HCBS). This issue further exacerbates sample attrition and may weaken the generalizability of the estimates. Therefore, the specification including community fixed effects is treated only as a robustness check rather than being incorporated into the baseline model.

Moreover, because the OLS and 2SLS models are subject to concerns regarding endogeneity and external validity, respectively, we primarily rely on the ATE estimated from the MTE model to capture the main effect of HCBS utilization on life satisfaction. Therefore, robustness checks for these two models are not reported in the table. Nevertheless, even after controlling for community fixed effects in these two models, the coefficient on HCBS utilization remains positive and statistically significant (OLS: *β* = 0.085, SE = 0.017, *p* < 0.01; 2SLS: *β* = 1.312, SE = 0.517, *p* < 0.05).

#### 3.5.7 Using clustered standard errors

Given that the study sample has a hierarchical structure in which individuals are nested within communities, and that the residuals of life satisfaction may exhibit intra-community correlation, we re-estimated the models using standard errors clustered at the community level. Panel (F) of Supplementary Table 3 shows that, after applying community-level clustered standard errors, the estimated ATE and ATT remain positive and statistically significant, whereas the ATUT remains statistically insignificant. Additionally, the *p*-values for both observable and essential heterogeneity remain statistically significant, thereby further supporting the baseline findings.

Moreover, it should also be noted that, although our data exhibit a nested structure, we employ heteroskedasticity-robust standard errors in the baseline models, while using community-clustered standard errors as a robustness check, for both methodological and applied reasons. First, recent methodological studies suggest that, within the causal inference context, the primary objective is typically to estimate treatment effect parameters. Under such contexts, residual autocorrelation induced by the nested data structure is neither a sufficient condition nor a necessary condition for cluster adjustment of standard errors. Moreover, in this context, the use of clustered standard errors may instead lead to unnecessarily conservative confidence intervals (75). Given that this study is exploratory in nature and aims to identify heterogeneous treatment effects of HCBS utilization on older adults’ life satisfaction, clustering standard errors in the baseline models may yield overly conservative confidence intervals, thereby potentially obscuring meaningful and policy-relevant heterogeneous findings. Therefore, we adopt a more flexible specification with heteroskedasticity-robust standard errors in the baseline models, which allows us to more fully capture potential observable and essential heterogeneity patterns in the data and leaves room for further exploration and validation in future studies. Second, from the perspective of existing empirical applications, studies that use individual-level HCBS utilization as the treatment variable to examine its impact on Chinese older adults’ life satisfaction and explicitly report the type of standard errors have typically employed heteroskedasticity-robust standard errors (2, 26, 27). In addition, as evidence in this field continues to accumulate, it has become increasingly important to synthesize existing findings through meta-analysis (16). Accordingly, by maintaining consistency with prior research in both variable operationalization and standard error specification, our study is able to maintain comparability with other related studies and facilitates the aggregation of results on a common scale in future meta-analytic work, thereby contributing to evidence-based decision-making for strengthening China’s HCBS system.

In addition, given that the OLS and IV models are subject to concerns regarding endogeneity and external validity, respectively, we do not report the robustness check results for these models in the table. Nevertheless, even after applying community-level clustered standard errors in these two models, the coefficient on HCBS utilization remains positive and statistically significant (OLS: *β* = 0.076, SE = 0.026, *p* < 0.01; 2SLS: *β* = 0.815, SE = 0.413, *p* < 0.05).

### 3.6 Mechanism analysis

To explain the formation of the positive selection pattern in the returns to HCBS utilization, we followed the approach of Jin and Liu (76) by estimating MTE specifications using each of the six mechanism variables as the outcome variable, thereby examining the underlying mechanisms within the framework of the bio-psycho-social model of successful aging (12).

To improve the clarity and interpretability of the mechanism analysis, we adopted a dimension-reduction idea within the MTE framework to identify which mechanisms drive our baseline MTE results (77, 78). Specifically, we first examined whether the returns to HCBS utilization exhibit a positive selection pattern across different mechanism variables by comparing the ATT and the ATUT. A positive selection pattern was considered to exist when the ATT exceeded the ATUT. We then assessed whether observable and unobservable characteristics jointly contributed to this pattern based on the statistical significance of the *p*-values for observable heterogeneity and essential heterogeneity, respectively. Finally, those mechanism variables whose positive selection patterns were jointly driven by observable and unobservable characteristics were identified as the primary drivers of positive selection on gains in life satisfaction.

Table 4 presents the results of the mechanism analysis. Columns (1) to (4) report the MTE estimates of HCBS utilization on physical and mental health. For SRH, the ATE is −0.235 (*p* > 0.1), the ATT is 1.007 (*p* < 0.01), and the ATUT is −1.320 (*p* < 0.01). For depression, the ATE is −4.675 (*p* < 0.01), the ATT is −8.874 (*p* < 0.01), and the ATUT is −1.208 (*p* > 0.1). However, for ADL and IADL, the ATE, ATT, and ATUT are not statistically significant. Column (5) reports the results for psychological resilience, with an ATE of 0.516 (*p* < 0.01), an ATT of 0.418 (*p* < 0.05), and an ATUT of 0.600 (*p* < 0.01). Similarly, Column (6) shows that the ATE, ATT, and ATUT for social participation are 2.781 (*p* < 0.01), 2.685 (*p* < 0.01), and 2.864 (*p* < 0.01), respectively. These findings indicate that HCBS utilization is more effective in improving SRH and reducing depression among older adults who are more likely to use HCBS, thereby exhibiting a pattern of positive selection on gains. However, the effects of HCBS utilization on the remaining four mechanism variables do not display a similar pattern. Specifically, HCBS utilization has no statistically significant effects on ADL or IADL across different groups of older adults. By contrast, HCBS utilization exerts significantly positive effects on psychological resilience and social participation across different groups, with the ATUT estimates being slightly larger than the ATT estimates, suggesting a pattern of negative selection on gains.

**Table 4:** Mechanism analysis.

Variables	SRH	ADL	IADL	Depression	Psychological resilience	Social participation
	(1)	(2)	(3)	(4)	(5)	(6)
ATE	−0.235 (0.265)	0.037 (0.233)	−0.431 (0.385)	−4.675^***^ (1.112)	0.516^***^ (0.144)	2.781^***^ (0.164)
ATT	1.007^***^ (0.302)	0.221 (0.253)	0.047 (0.427)	−8.874^***^ (1.308)	0.418^**^ (0.186)	2.685^***^ (0.197)
ATUT	−1.320^***^ (0.418)	−0.124 (0.373)	−0.848 (0.611)	−1.208 (1.705)	0.600^***^ (0.214)	2.864^***^ (0.253)
Observable heterogeneity	<0.01	<0.01	<0.01	<0.01	<0.01	<0.01
Essential heterogeneity	<0.01	0.44	0.23	<0.01	0.50	0.62

^*^p < 0.1, ^**^p < 0.05, ^***^p < 0.01. Robust standard errors are reported in parentheses. All models include control variables and province fixed effects; corresponding coefficients are omitted to save space. The observable heterogeneity column reports the p-values from joint significance tests of β_1_-β_0_ coefficients for observable characteristics in models where different mechanism variables are used as dependent variables. Statistically significant p-values in this column indicate that the effect of HCBS utilization on the corresponding mechanism variables varies with observable characteristics. The essential heterogeneity column reports the p-values from tests of whether the slope of the MTE curve equals zero in models with different mechanism variables as dependent variables. Statistically significant *p*-values in this column indicate that the effect of HCBS utilization on the corresponding mechanism variables varies with unobserved characteristics.

Moreover, the *p*-values for observable heterogeneity in Columns (1) to (6) are all statistically significant. In contrast, the p-values for essential heterogeneity are statistically significant only in Columns (1) and (4). These results suggest that both observable and unobservable characteristics jointly drive the heterogeneous effects of HCBS utilization on SRH and depression among older adults. Taken together, these findings imply that the heterogeneous effects of HCBS utilization on life satisfaction can be largely explained by its heterogeneous impacts on SRH and depression.

### 3.7 Policy simulations

The above analysis indicates that older adults who are more likely to use HCBS gain greater benefits from it, suggesting that policies aimed at reducing barriers to HCBS utilization could yield substantial gains. Accordingly, we quantify the associated returns for six different policy scenarios, as presented in Table 5.

**Table 5:** Policy simulations.

Policy scenarios	HCBS utilization rate		
	Baseline	Policy	PRTE
	(1)	(2)	(3)
(1) Raise the HCBS utilization rate by 5%	46.7%	51.6%	0.571^**^ (0.257)
(2) Raise the HCBS utilization rate by 10%	46.7%	56.6%	0.489^*^ (0.263)
(3) Increase the coverage rate of community service facilities to 100%	46.7%	48.2%	0.878^***^ (0.248)
(4) Increase the coverage rate of pension insurance to 100%	46.7%	47.2%	0.804^***^ (0.253)
(5) Increase the coverage rate of medical insurance to 100%	46.7%	47.4%	0.663^**^ (0.264)
(6) Increase the coverage rates of community service facilities, pension insurance, and medical insurance to 100%	46.7%	49.7%	0.738^***^ (0.251)

^*^p < 0.1, ^**^p < 0.05, ^***^p < 0.01. Robust standard errors in parentheses.

We begin by simulating two policies that directly increase the HCBS utilization rate (or, equivalently, the propensity score) from its baseline level of 46.7% by 5 and 10 percentage points, respectively. Rows (1) and (2) show that the corresponding PRTEs are 0.571 (*p* < 0.05) and 0.489 (*p* < 0.1), respectively, indicating that both policies significantly improve life satisfaction among new HCBS users.

However, directly manipulating the propensity score does not provide practical guidance on how to increase the HCBS utilization rate. Therefore, based on the results from the MTE estimation, we simulate four policies that indirectly raise HCBS utilization, including: (i) increasing the coverage rate of community service facilities to 100%; (ii) increasing the coverage rate of pension insurance to 100%; (iii) increasing the coverage rate of medical insurance to 100%; and (iv) simultaneously increasing the coverage rates of all three to 100%. Rows (3) to (6) show that these policies raise the HCBS utilization rate from the baseline level of 46.7 to 48.2%, 47.2, 47.4, and 49.7%, respectively, with corresponding PRTEs of 0.878 (*p* < 0.01), 0.804 (*p* < 0.01), 0.663 (*p* < 0.05), and 0.738 (*p* < 0.01). These results suggest that the four policies can increase HCBS utilization rate by 0.5 to 3 percentage points and significantly enhance life satisfaction among new HCBS users.

## 4 Discussion

This study draws on nationally representative data on older adults in China and applies the MTE framework to examine the causal and heterogeneous effects of HCBS utilization on life satisfaction, explore the mechanisms underlying treatment effect heterogeneity, and simulate the expected returns of policies aimed at increasing HCBS uptake.

First, our study demonstrates that HCBS utilization significantly enhances life satisfaction among older adults. This finding aligns with previous evidence drawn from samples in developed countries such as the United States, Canada, and South Korea (16–18). Meanwhile, our study contributes to the literature examining the relationship between HCBS utilization and older adults’ life satisfaction in the Chinese context. Specifically, empirical evidence based on Chinese data remains inconclusive. Although most studies report a positive association between HCBS utilization and life satisfaction, some reach contrasting conclusions. For example, Cheung et al. (7), using survey data from Hong Kong in 2002, find that the use of meal delivery and medical services is significantly negatively associated with older adults’ quality of life. Lu et al. (31), using data from the Chinese Longitudinal Healthy Longevity Survey (CLHLS) from 2008 to 2014, report that unmet service needs resulting from discrepancies between community service provision and older adults’ demands significantly predict lower baseline life satisfaction. However, these studies rely on relatively early data, when HCBS development in China was still in its initial stage and the availability, quality, and accessibility of services were limited, potentially failing to meet older adults’ needs and thereby undermining life satisfaction. In contrast, studies using more recent data generally find that HCBS utilization is significantly associated with higher life satisfaction among older adults (2, 6, 14, 23, 26–30, 48). Although the present study is consistent in qualitative terms with this latter strand of the literature, it makes important methodological contributions. Specifically, some studies rely on multivariate regression or matching methods (6, 14, 26–29), which can only address selection bias arising from observable characteristics; these approaches may still yield biased estimates in the presence of unobserved confounders. Some studies employ panel data and fixed effects models to eliminate time-invariant unobserved heterogeneity, but such approaches may still suffer from time-varying omitted variable bias (2, 23, 30). Other studies adopt an IV approach to address endogeneity concerns (48); however, the validity of the IV—particularly the exclusion restriction—is often not fully examined, which may undermine the credibility of the estimates. Moreover, IV estimates identify the LATE for compliers, which limits the external validity of the findings. In contrast, we rigorously assess the validity of the IV using advanced econometric methods and conduct extensive robustness checks to evaluate the sensitivity of the estimates. These efforts help to rule out potential threats to identification and strengthen the credibility of our results. In addition, we extrapolate IV estimates to the full sample within the MTE framework to obtain the ATE, thereby providing more generalizable causal evidence that HCBS utilization improves older adults’ life satisfaction. Our ATE estimates suggest that HCBS utilization increases older adults’ life satisfaction by 14.1% on average. This substantial effect can be interpreted from multiple perspectives. Specifically, HCBS can address functional needs, promote psychological adaptation, and improve the fit between environmental demands and individual competence, thereby increasing happiness (17, 26). In addition, HCBS can substitute for informal care provided by family members, alleviating household resource constraints and caregiving burdens, and reducing older adults’ unmet care needs, thereby improving their quality of life (7). Moreover, HCBS helps lower physical barriers and environmental hazards, promoting age-friendly communities that support aging in place and enhance older adults’ social and mental well-being (13).

Second, this study finds substantial heterogeneity in the impact of HCBS utilization on life satisfaction among older adults with respect to both observed and unobserved characteristics. Older adults who are female, of advanced age, from ethnic minority groups, and enrolled in pension and medical insurance are not only more likely to use HCBS but also experience higher returns in terms of life satisfaction, indicating a positive selection on returns based on observed characteristics. A plausible explanation is that older adults who are female, of advanced age, and from ethnic minority groups are often socioeconomically disadvantaged and have limited access to institutional care. HCBS is more affordable and accessible, and thus better aligns with their financial constraints and care needs, resulting in greater benefits (52). Moreover, in China, older adults enrolled in pension and medical insurance are eligible for long-term care insurance and associated HCBS subsidies, which further increases both their likelihood of utilization and the resulting gains. Selection on unobserved characteristics reinforces this finding: older adults with unobserved characteristics that predispose them to use HCBS benefit the most from HCBS utilization, whereas those least likely to use such services benefit the least. Consequently, the ATT exceeds the ATUT, with ATT being strongly positive and statistically significant, and ATUT being negative and statistically insignificant. The positive selection on returns highlights the importance of person–environment fit: HCBS, as a component of the community support system, is more likely to enhance life satisfaction when it aligns with older adults’ needs and characteristics (32). Furthermore, this positive selection pattern also reflects older adults’ preventive-corrective proactivity, that is, when exposed to stressors such as economic disadvantage and health decline, they can leverage available resources to build resilience and mitigate adverse impacts, thereby improving their life satisfaction (79). In addition, we move beyond a binary indicator of HCBS utilization (i.e., whether individuals use HCBS) and further examine the effects of the category of HCBS utilization and the number of services utilized on older adults’ life satisfaction. The results indicate that, first, the effects of both subtypes of HCBS (including medical services and eldercare services) on life satisfaction exhibit a pattern of positive selection on gains. Second, the positive selection pattern in returns to HCBS utilization is primarily concentrated at the extensive margin (i.e., no HCBS utilization vs. any HCBS utilization), rather than at the intensive margin (i.e., some HCBS utilization vs. more HCBS utilization). These findings not only validate the credibility of treating HCBS utilization as an integrated construct in terms of variable operationalization, but also highlight the policy relevance of prioritizing interventions aimed at increasing HCBS uptake. Nevertheless, the ATE estimates regarding the category of HCBS utilization suggest that medical services may be the primary driver of the positive and statistically significant average effects of HCBS utilization on older adults’ life satisfaction. One possible explanation is that, although China’s HCBS system is designed to promote an integrated model of medical and eldercare services, traditional cultural norms emphasizing filial piety mean that family-based eldercare remains the predominant choice for most older adults. As a result, the utilization rate of eldercare services is substantially lower than that of medical services. From a methodological perspective, the relatively low utilization rate of eldercare services implies that the associated benefits are concentrated among a small group of high-propensity individuals, thereby highlighting a pattern of positive selection on gains and making the overall ATE statistically insignificant. However, given that the ATT of eldercare services on life satisfaction is positive and statistically significant, it can be expected that their overall returns would become more pronounced as the utilization rate increases. Another potential explanation is that medical services are more specialized and cannot be readily substituted by informal care provided within the household. In addition, HCBS medical services are characterized by greater accessibility and affordability, which enables a larger proportion of older adults to rely on and benefit from them more substantially, thereby generating a significantly positive ATE. Moreover, the positive effects of medical services are consistent with the results of the mechanism analysis, which show that the heterogeneous effects of HCBS utilization on life satisfaction are primarily driven by heterogeneity in health returns. Taken together, these findings provide a form of complementary evidence, further underscoring the role of medical services provided under the HCBS system and their potentially beneficial health impacts in improving older adults’ life satisfaction.

Third, we conducted an exploratory mechanism analysis based on the bio-psycho-social model of successful aging and found that the heterogeneous effects of HCBS utilization on older adults’ life satisfaction are primarily driven by the heterogeneity in its health returns. Specifically, HCBS utilization is more effective in improving SRH and reducing depression among those with a higher propensity to use such services. Given the well-established role of health in enhancing life satisfaction among older adults, these findings suggest that SRH and depression may mediate the heterogeneous impact of HCBS utilization on life satisfaction. Previous studies have demonstrated that HCBS can improve older adults’ physical and mental health—and thereby life satisfaction—by offering therapeutic and rehabilitative care, facilitating the dissemination of health information, and providing emotional support (11, 14, 16). Our results further indicate that the mediating roles of SRH and depression are likely unconditional, operating across the entire distribution of propensity scores rather than being limited to specific subgroups. However, we do not observe statistically significant effects of HCBS utilization on ADL or IADL across any group. One possible explanation is that impairments in ADL and IADL are highly irreversible, while China’s HCBS system is characterized by its basic and universal nature, making it difficult to meet more specialized medical needs. As a result, HCBS is insufficient to substantially slow the natural decline in physical functioning among older adults, and even less able to reverse functional impairments among disabled older adults (14). Nevertheless, HCBS utilization does not exert any statistically significant negative effects on ADL or IADL among older adults, which may suggest that service components within the HCBS system—such as rehabilitation training and the rental of rehabilitative assistive devices—can meet the rehabilitation needs of disabled older adults, thereby helping to maintain their physical functioning. Additionally, we found that HCBS utilization significantly improves psychological resilience and social participation across all groups of older adults, indicating that the psychological and social pathways cannot account for the pattern of positive selection in life satisfaction gains. This finding suggests that, although HCBS may not improve the physical and mental health of older adults with low utilization propensity, it may provide them with opportunities to activate adaptive psychological and social mechanisms, thereby supporting their autonomy and engagement (79). Nevertheless, the baseline MTE specification yielded a statistically insignificant estimate for the ATUT, indicating that HCBS utilization did not significantly improve life satisfaction among the low-propensity group. This finding implies that the improvements in psychological resilience and social participation associated with HCBS utilization were insufficient to translate into gains in life satisfaction for this group. One possible explanation is provided by our mechanism analysis, which showed that the ATUT estimate of HCBS utilization on SRH was negative and statistically significant, suggesting that service utilization may exacerbate unmet health needs among the low-propensity group due to mismatches between service provision and actual healthcare demands, thereby leading them to downgrade their subjective perceptions of health status. As a result, the decline in SRH experienced after HCBS utilization may offset the gains in psychological resilience and social participation, ultimately preventing significant improvements in life satisfaction. In addition, constrained by data availability, our empirical analysis was unable to incorporate all potential mechanism outcomes. Therefore, other unconsidered factors may still exist in the relationship between HCBS utilization and life satisfaction, which could explain why the low-propensity group did not obtain significant returns from HCBS utilization. To address this limitation, future research should incorporate a broader range of potential mechanism variables to achieve a more comprehensive understanding of the factors driving the returns to HCBS utilization among the low-propensity group and to further enhance the positive role of the HCBS system in improving life satisfaction among older adults.

Finally, we simulated the expected effects of policies aimed at increasing HCBS utilization. Given that informal family care remains the dominant form of elder care in China—particularly under the government’s “9,073” care model, which envisions that around 90% of older adults rely on informal family care, 7% receive community-based care, and 3% reside in institutions—we first simulated the expected effects of a moderate increase in the HCBS utilization rate by 5 and 10%. We found that HCBS utilization significantly improves life satisfaction among new users. This finding suggests that the current number of HCBS users is lower than the number of older adults who could potentially benefit from such services. To explore how the HCBS utilization rate might be increased, we simulated the policy impacts of expanding the coverage of community service facilities, pension insurance, and medical insurance to 100%, either individually or simultaneously, in line with the objectives advocated by multiple policy guidelines issued by the Chinese government. We found that these policies lead to significant gains in life satisfaction; however, they only increase the overall HCBS utilization rate by 0.5 to 3 percentage points. These findings may suggest a concave relationship between the coverage rates of these programs and the HCBS utilization rate. Given that the existing coverage rates of community service facilities, pension insurance, and medical insurance are already high (80.9, 86.8, and 95.4%, respectively), even full coverage is unlikely to produce substantial improvements in HCBS uptake. Therefore, additional measures are necessary to further incentivize HCBS utilization among older adults. These may include reimbursement schemes or subsidy programs aimed at lowering the cost of HCBS utilization, as well as initiatives to expand service scope and enhance service quality to better meet the multiple needs of older adults. Moreover, because of China’s strong cultural emphasis on filial piety, older adults and their families may face psychological and social barriers to HCBS utilization due to stigma. Thus, public awareness campaigns informing these communities about the benefits of HCBS are also essential. In sum, policymakers should reduce barriers and promote HCBS utilization to maximize its potential in improving older adults’ life satisfaction.

This study makes several key contributions. First, at the theoretical level, this study extends the analytical perspective of the existing literature by adopting the MTE framework and deepens the understanding of heterogeneous returns to HCBS utilization. Specifically, the person–environment fit perspective in gerontology emphasizes that older adults’ well-being is the outcome of the interaction between personal competence and environmental support. The empirical literature has also widely acknowledged that the effects of HCBS utilization on older adults’ life satisfaction are heterogeneous. However, most studies explore treatment effect heterogeneity through subgroup analyses or interaction-term approaches, which can capture only heterogeneity driven by observable characteristics while largely neglecting unobserved sources of heterogeneity (11, 23, 26–30). To address this limitation, we introduce the MTE framework to explicitly account for treatment effect heterogeneity conditional on both observed and unobserved characteristics, and we summarize heterogeneity across different dimensions based on how returns vary with the propensity to use HCBS. We document a pattern of positive selection on gains and further show that heterogeneous health returns constitute the main mechanism driving this pattern. These findings provide a more comprehensive picture of the heterogeneous returns to HCBS utilization, advance the theoretical interpretation of person–environment fit, and offer a new analytical perspective for future research examining heterogeneous effects of HCBS on other dimensions of well-being outcomes. Second, at the methodological level, this study provides more credible and generalizable causal evidence on the effect of HCBS utilization on older adults’ life satisfaction. Specifically, we employ an IV approach to address potential endogeneity concerns and conduct quantitative tests to assess the validity of the IV. Moreover, we apply the MTE framework for extrapolation, thereby achieving a better balance between internal validity and external validity and enhancing the generalizability of the estimated results. In particular, we construct the key treatment variable as an individual-level binary indicator of HCBS utilization. This approach is consistent with the majority of existing studies and helps improve the comparability of our findings with the broader literature, thereby facilitating the accumulation of evidence in this field. Meanwhile, compared with studies that use regional HCBS pilot policies as treatment variables (23), our approach more precisely captures micro-level policy effects, thereby providing empirical support for a more nuanced quantitative understanding of the subjective well-being returns to HCBS utilization. Finally, at the practical level, this study offers policy implications for strengthening the positive impact of HCBS on older adults’ life satisfaction. In particular, we estimate PRTEs and simulate the expected effects of policies aimed at increasing HCBS uptake, moving beyond prior studies that have primarily focused on ex post evaluations of the relationship between HCBS utilization and life satisfaction (16). Given the rapid population aging in China and the accelerated expansion of HCBS, these simulation results provide valuable policy insights for optimizing HCBS system design and improving implementation effectiveness.

Several limitations should be acknowledged. First, since HCBS did not cover all provinces in China until 2021, HCBS was only available in specific policy pilot regions in the earlier waves of the CLASS survey. At the same time, as the content of HCBS has expanded over time, the specific HCBS items covered in earlier waves of the CLASS questionnaire differed substantially, whereas the 2023 wave includes the most comprehensive set of HCBS measures. Therefore, we use the 2023 CLASS data to ensure the timeliness and representativeness of the analysis. In addition, although panel data can control for time-invariant confounding factors, the presence of time-varying omitted variables would still require causal inference strategies, such as the IV approach, to address endogeneity. Moreover, a key objective of this study is to examine the heterogeneous effects of HCBS utilization. When using panel data, time-invariant characteristics, such as gender, ethnicity, pension insurance, and medical insurance, which drive the pattern of positive selection in gains, would be absorbed by individual fixed effects, thereby obscuring these important sources of heterogeneity. Therefore, after confirming the validity of the IV, combining the 2023 CLASS cross-sectional data with IV and MTE approaches allows us to credibly identify causal effects and uncover the potential drivers of heterogeneous effects. Nevertheless, the cross-sectional nature of the data limits our analysis to the short-term effects of HCBS utilization and prevents us from examining its long-term impacts. Future research could incorporate subsequent waves of the CLASS data to trace changes in life satisfaction among HCBS users over time. Second, while we identify biological pathways as a plausible mechanism underlying the heterogeneous effects of HCBS, the lack of data on physiological constitution and biomarkers prevents us from directly testing this channel. Future research could collect such biological measures to better understand the role of biological pathways in shaping heterogeneous effects. Third, due to data availability constraints, we only examine three policy interventions aimed at increasing HCBS utilization, namely expanding the coverage of community service facilities, pension insurance, and medical insurance. As a result, it is difficult to incorporate supply-side reform measures, such as reducing service prices and improving service quality, or policy initiatives designed to alleviate the cultural and psychological barriers that discourage older adults from using HCBS and to enhance their willingness to use such services. In particular, given that expanding the coverage of community service facilities or social insurance alone may be insufficient to substantially increase HCBS utilization, these alternative policy interventions that are not considered in the present study may in fact hold greater potential. Therefore, future research should further investigate how factors such as service price, service quality, and older adults’ awareness of available services influence HCBS utilization, and examine how corresponding policy interventions affect the returns to these services. Moreover, it should be noted that the external validity of the PRTE estimates relies heavily on model assumptions. Although the robustness checks provide statistical evidence supporting the appropriateness of the MTE model specification, the policy simulation results based on the PRTE should still be interpreted as suggestive rather than conclusive. Accordingly, our simulation results can only provide policymakers with preliminary insights into potential strategies for optimizing HCBS, and future research using real world data is still needed to validate the effectiveness of these policy interventions.

## 5 Conclusion

This study examines the impact of HCBS utilization on life satisfaction among older adults in China. Applying the MTE framework, we address the endogeneity of HCBS utilization, enhance the external validity of the estimated treatment effects, and account for both observable and essential heterogeneity in treatment effects. Our analyses show that, on average, HCBS utilization can enhance life satisfaction. However, older adults who benefit the most from HCBS are the most likely to use HCBS, indicating a pattern of positive selection on gains. Furthermore, we confirm that heterogeneous health returns serve as a crucial mechanism underlying this pattern. These results offer new insights into the complex relationship between HCBS utilization and life satisfaction among older adults and raise a critical question: what types of policies can reduce barriers to HCBS utilization to ensure that a broader older population can benefit? In response, we conduct additional policy simulations and find that increasing HCBS utilization rates can improve life satisfaction among new users. In particular, expanding the coverage of community service facilities, pension insurance, and medical insurance can indirectly enhance life satisfaction by promoting HCBS utilization, although the magnitude of their effects on HCBS uptake appears modest.

Our findings hold important policy implications. Specifically, our main effect results suggest that increasing HCBS coverage is generally associated with higher life satisfaction among older adults. The policy simulation results not only provide further support for this finding but also demonstrate the potential effectiveness of policy interventions such as expanding the coverage of community service facilities, pension insurance, and medical insurance. Theoretically, these three types of interventions considered in our simulations help improve the accessibility of HCBS and increase older adults’ access to service-related subsidies. However, given that the current coverage rates of community service facilities and social insurance are already relatively high, their marginal effects on HCBS uptake are limited. These findings suggest that governments should adopt more diversified policy interventions, such as reducing service costs, expanding service offerings, improving service quality, and strengthening policy communication to enhance older adults’ preferences for HCBS, thereby further strengthening the role of HCBS in improving older adults’ quality of life. In addition, the results from the heterogeneity and mechanism analyses indicate that the returns to HCBS utilization are unequally distributed across older adults, and that heterogeneous health returns constitute the primary driver of this pattern. These findings imply that governments should enhance the inclusiveness and equity of community health service provision, increase fiscal support for economically disadvantaged regions, and cultivate a more professional general practitioner workforce, thereby delivering higher-quality and more specialized health services. Such efforts would help meet the diverse health needs of older adults, reduce unmet health care needs, and ultimately improve the overall health and well-being of the older population.

## Data Availability

Publicly available datasets were analyzed in this study. This data can be found at: http://class.ruc.edu.cn/.

## References

[1] United Nations World Population Prospects 2024. New York: United Nations (2024).

[2] Song H Li Z. Community-based service, psychological resilience and life satisfaction among Chinese older adults: a longitudinal study. Geriatr Nurs. (2023) 54:148–54. doi: 10.1016/j.gerinurse.2023.09.004, 37788562

[3] Wang H Liu H Wu B Hai L. The association between trajectories of perceived unmet needs for home and community-based services and life satisfaction among Chinese older adults: the moderating effect of psychological resilience. Res Aging. (2024) 46:139–52. doi: 10.1177/01640275231203608, 37768843

[4] Jiang WK Kuang JF Gan KP. Internet use and life satisfaction among Chinese middle-aged and older adults: the role of volunteering and social trust. J Gerontol Soc Work. (2025) 69:458–74. doi: 10.1080/01634372.2025.2515603, 40485077

[5] Diener E. Subjective well-being. Psychol Bull. (1984) 95:542–75. 6399758

[6] Jiang C Chow JCC Zhou L Song H Shi J. Community support, social isolation and older adults’ life satisfaction: evidence from a national survey in China. Aging Ment Health. (2023) 28:849–57. doi: 10.1080/13607863.2023.2277871, 37921357

[7] Cheung JCK Kwan AYH Chan SSC Ngan RMH Ng SH Leung EMF . Quality of life in older adults: benefits from caring services in Hong Kong. Soc Indic Res. (2005) 71:291–334. doi: 10.1007/s11205-004-8021-3

[8] Khodabakhsh S. Factors affecting life satisfaction of older adults in Asia: a systematic review. J Happiness Stud. (2022) 23:1289–304. doi: 10.1007/s10902-021-00433-x

[9] Spruk R Kešeljević A. Institutional origins of subjective well-being: estimating the effects of economic freedom on national happiness. J Happiness Stud. (2016) 17:659–712. doi: 10.1007/s10902-015-9616-x

[10] Sen Q Lei Z. The impact of community care services on older people’s psychological health: an empirical study in Liaoning province, China. Front Public Health. (2023) 11:1199830. doi: 10.3389/fpubh.2023.1199830, 37601200 PMC10436539

[11] Su Q Wang H Fan L. The impact of home and community care services pilot program on healthy aging: a difference-in-difference with propensity score matching analysis from China. Arch Gerontol Geriatr. (2023) 110:104970. doi: 10.1016/j.archger.2023.104970, 36842402

[12] Kanning M Schlicht W. A bio-psycho-social model of successful aging as shown through the variable “physical activity”. Eur Rev Aging Phys Act. (2008) 5:79–87. doi: 10.1007/s11556-008-0035-4

[13] Au A Lai DWL Yip H Chan S Lai S Chaudhury H . Sense of community mediating between age-friendly characteristics and life satisfaction of community-dwelling older adults. Front Psychol. (2020) 11:86. doi: 10.3389/fpsyg.2020.00086, 32194465 PMC7064721

[14] Gu S Jia C Shen F Wang X Wang X Gu H. The effects of community-based home health care on the physical and mental health of older adults with chronic diseases. Qual Life Res. (2024) 33:691–703. doi: 10.1007/s11136-023-03555-2, 38032396

[15] Ni C Li M Pan W Wang Z. The impact of integrated medical and nursing care on elderly health: mechanisms and empirical evidence. China J Econ. (2025) 12:228–46.

[16] Lyu X Fan Y. The impact of home- and community-based services on the health of older adults: a meta-analysis. SAGE Open. (2024) 14:14. doi: 10.1177/21582440241285674, 41092927

[17] Kadowaki L Wister AV Chappell NL. Influence of home care on life satisfaction, loneliness, and perceived life stress. Can J Aging. (2015) 34:75–89. doi: 10.1017/S0714980814000488, 25547720

[18] Park S Lee S. Age-friendly environments and life satisfaction among south Korean elders: person–environment fit perspective. Aging Ment Health. (2016) 21:693–702. doi: 10.1080/13607863.2016.1154011, 26938196

[19] Li Q Xiao Y Liu H Zhao C. Public elderly care services and labor supply: evidence from the home- and community-based elderly care services pilot. Econ Res J. (2024) 59:186–202.

[20] Liu Z Zhang Y. Can “care for the elderly” promote “offspring’s employment”—impact of community home-based elderly care service on offspring’s off-farm employment. J Shanxi Univ Financ Econ. (2024) 46:69–81.

[21] Xu K Li J Deng H. Intergenerational economic interaction and the vision of “home-based elderly care”: evidence from China's pilot reform of elderly care services. Chin Rev Financ Stud. (2024) 16:84–100+152-153.

[22] Song Y Gao Y Zhan J. Can public elderly care services alleviate the filial support burden on adult children: evidence from the pilot reform of home-and community-based elderly care services. Soc Secur Stud. (2025) 6:20–39.

[23] Lu B Chen N. Does home- and community-based care improve older adults’ subjective well-being? Evidence from a quasi-natural experiment of the pilot policy on home- and community-based care services. J Zhengzhou Univ (Philos Soc Sci Ed). (2024) 57:18–24.

[24] Jiang H Huang S Wang Z. The impact of home-based community elderly care pilot reforms on silver-haired consumption. Popul Econ. (2025) 5:14–30. doi: 10.3969/j.issn.1000-4149.2025.05.002

[25] Li L Peng D. Reform of home and community-based elderly care services and improvement of elderly consumers’ welfare. Consum Econ. (2025) 41:106–20.

[26] Liu Y Yang S. The impact of community older adult care services on the quality of life of older adults in China: the mediating role of social adaptation. Front Public Health. (2025) 13:1544575. doi: 10.3389/fpubh.2025.1544575, 40365436 PMC12069073

[27] Ma W Zheng X Fang X. The influence of community endowment service on life satisfaction of the elderly: a mediating effect analysis based on physical health and leisure activity. J South China Univ Technol (Soc Sci Ed). (2019) 21:94–107.

[28] Zhang Z Mao Y Shui Y Deng R Hu Y. Do community home-based elderly care services improve life satisfaction of Chinese older adults? An empirical analysis based on the 2018 CLHLS dataset. Int J Environ Res Public Health. (2022) 19:15462. doi: 10.3390/ijerph192315462, 36497536 PMC9738417

[29] Jia CC Zhang L Wang ZY Li Z. Life satisfaction among older adults in China: who gains more from community-based health services? Front Public Health. (2025) 13:1705076. doi: 10.3389/fpubh.2025.1705076, 41426657 PMC12715604

[30] Sun Z. Home-based elder care services and older adults’ life satisfaction: evidence from the China longitudinal aging social survey. J Soc Dev. (2025) 12:206–225+246.

[31] Lu P Shelley M Kong D. Unmet community service needs and life satisfaction among Chinese older adults: a longitudinal study. Soc Work Public Health. (2021) 36:665–76. doi: 10.1080/19371918.2021.1948942, 34340642

[32] Chen Q Amano T Park S Kim B. Home and community-based services and life satisfaction among homebound and poor older adults. J Gerontol Soc Work. (2019) 62:708–27. doi: 10.1080/01634372.2019.1639094, 31293224

[33] Wu J Chen D Li C Wang Y. Effect of community-based public health service on health-related quality of life among middle-aged and older adults with chronic diseases in China. BMC Public Health. (2024) 24:2039. doi: 10.1186/s12889-024-19556-w, 39080595 PMC11290236

[34] Yang L Wang L Dai X. Rural-urban and gender differences in the association between community care services and elderly individuals’ mental health: a case from Shaanxi Province, China. BMC Health Serv Res. (2021) 21:106. doi: 10.1186/s12913-021-06113-z, 33516212 PMC7847576

[35] Wahl HW Iwarsson S Oswald F. Aging well and the environment: toward an integrative model and research agenda for the future. Gerontologist. (2012) 52:306–16. doi: 10.1093/geront/gnr154, 22419248

[36] Wahl HW Gerstorf D. Person–environment resources for aging well: environmental docility and life space as conceptual pillars for future contextual gerontology. Gerontologist. (2020) 60:368–75. doi: 10.1093/geront/gnaa006, 32240292

[37] Chen M Bolt G Hooimeijer P. The impact of residential environment on older people's capabilities to live independently: a survey in Beijing. BMC Public Health. (2024) 24:843. doi: 10.1186/s12889-024-18262-x, 38500091 PMC10949666

[38] Zhou JJ Zhou S Cai XX Luo JM. Longitudinal associations between neighborhood environments and functional disabilities among community-dwelling older adults in China. J Gerontol B Psychol Sci Soc Sci. (2025) 80:gbae206. doi: 10.1093/geronb/gbae206, 39812409

[39] Chaudhury H Oswald F. Advancing understanding of person-environment interaction in later life: one step further. J Aging Stud. (2019) 51:100821. doi: 10.1016/j.jaging.2019.100821, 31761094

[40] Lv X Zhang X. The influence of community home-based elderly care on the health of the elderly population. Chin J Popul Sci. (2022) 3:111–125+128.

[41] Jia K Xia Y Zhao G. Elderly care services and mental health: evidence from the pilot policies of home and community care services for the elderly in China. J World Econ. (2023) 46:163–85. doi: 10.19985/j.cnki.cassjwe.2023.08.007

[42] Yan SB. The impact of home and community-based services on the health status of older adults in China: a longitudinal analysis. Innov Aging. (2025) 9:igaf122.2839. doi: 10.1093/geroni/igaf122.2839

[43] Liu Z Liu L. How reforms in home-based and community elderly care service drive “successful” aging: difference-in-differences evidence and mechanism analysis based on pilot policies. Mod Financ Econ J Tianjin Univ Financ Econ. (2026) 46:113–37.

[44] Li J Shi M Zhou W. Research on the impact of community education services on social participation of elderly people living alone. Chin Health Serv Manag. (2025) 42:858–861+872.

[45] Blattman C Fiala N Martinez S. Generating skilled self-employment in developing countries: experimental evidence from Uganda. Q J Econ. (2014) 129:697–752. doi: 10.1093/qje/qjt057

[46] Shi J Zhang D Jiang C Wu Y Che R. Can smart home devices promote older adults’ subjective markers of successful aging? A cohort study exploring potential mechanisms. Geriatr Nurs. (2025) 65:103517. doi: 10.1016/j.gerinurse.2025.103517, 40628102

[47] Burke RE Xu Y Ritter AZ Werner RM. Postacute care outcomes in home health or skilled nursing facilities in patients with a diagnosis of dementia. Health Serv Res. (2022) 57:497–504. doi: 10.1111/1475-6773.13855, 34389982 PMC9108056

[48] Zhang W Su M Li D Yang F Li Z. The association between family doctor contract services and the health of middle-aged and older people in China: an instrumental variables analysis. Sci Rep. (2024) 14:16229. doi: 10.1038/s41598-024-65621-0, 39004624 PMC11247085

[49] Suzhou Industrial Park Administration Committee Improving the “10-Minute Elderly Care Service Circle”: The Donggang Second Community Daycare Center in Loufeng Subdistrict Officially Opens (2024). Available online at: https://www.sipac.gov.cn/szgyyq/dthg202401/202401/e860ee3722b44f268ceb87fc739cad58.shtml (Accessed May 21, 2026).

[50] Shenqiu County People’s Government Huadian Town Jinghua Community: Focusing Efforts on “Five-Star” Elderly Care Services to Build a Happy and Harmonious Community (2023). Available online at: https://www.shenqiu.gov.cn/newslast_37547.html (Accessed May 21, 2026).

[51] People’s Government of Tacheng Prefecture Hoboksar County: Community Daycare Centers Make Elderly Care a More Enjoyable Experience (2024). Available online at: https://www.xjtc.gov.cn/ywdt/jrtc/xsdt/content_46562 (Accessed May 21, 2026).

[52] Che RP Cheung MC. Factors associated with the utilization of home and community-based services (HCBS) among older adults: a systematic review of the last decade. J Gerontol Soc Work. (2024) 67:776–802. doi: 10.1080/01634372.2024.2342455, 38616618

[53] Ye J Zhang X. Relationship between resilience and well-being in elders: a systematic review and meta-analysis. Adv Psychol Sci. (2021) 29:202–17. doi: 10.3724/SP.J.1042.2021.00202

[54] Brinch CN Mogstad M Wiswall M. Beyond LATE with a discrete instrument. J Polit Econ. (2017) 125:985–1039. doi: 10.1086/692712, 2096062

[55] Heckman JJ Vytlacil E. "Econometric evaluation of social programs". In: Heckman JJ Leamer EE, editors. Part II Handbook of Econometrics. Amsterdam: Elsevier (2007). p. 4875–5143.

[56] Heckman JJ Lochner LJ Todd PE. "Earnings functions, rates of return and treatment effects: the mincer equation and beyond". In: Hanushek E Welch F, editors. Handbook of the Economics of Education. Amsterdam: Elsevier (2006). p. 307–458.

[57] Carneiro P Heckman JJ Vytlacil EJ. Estimating marginal returns to education. Am Econ Rev. (2011) 101:2754–81. doi: 10.1257/aer.101.6.2754, 25110355 PMC4126808

[58] Cornelissen T Dustmann C Raute A Schönberg U. Who benefits from universal child care? Estimating marginal returns to early child care attendance. J Polit Econ. (2018) 126:2356–409. doi: 10.1086/699979

[59] Sarr M Ayele MB Kimani ME Ruhinduka R. Who benefits from climate-friendly agriculture? The marginal returns to a rainfed system of rice intensification in Tanzania. World Dev. (2021) 138:105160. doi: 10.1016/j.worlddev.2020.105160

[60] Heckman JJ Vytlacil E. Structural equations, treatment effects, and econometric policy evaluation1. Econometrica. (2005) 73:669–738. doi: 10.1111/j.1468-0262.2005.00594.x

[61] Terza JV Basu A Rathouz PJ. Two-stage residual inclusion estimation: addressing endogeneity in health econometric modeling. J Health Econ. (2008) 27:531–43. doi: 10.1016/j.jhealeco.2007.09.009, 18192044 PMC2494557

[62] Ferrer-i-Carbonell A Frijters P. How important is methodology for the estimates of the determinants of happiness? Econ J. (2004) 114:641–59. doi: 10.1111/j.1468-0297.2004.00235.x, 40046247

[63] Kitagawa T. A test for instrument validity. Econometrica. (2015) 83:2043–63. doi: 10.3982/ECTA11974

[64] Mourifié I Wan Y. Testing local average treatment effect assumptions. Rev Econ Stat. (2017) 99:305–13. doi: 10.1162/REST_a_00622

[65] Chernozhukov V Lee S Rosen AM. Intersection bounds: estimation and inference. Econometrica. (2013) 81:667–737. doi: 10.3982/ECTA8718, 29959179

[66] Barron K Kung E Proserpio D. The effect of home-sharing on house prices and rents: evidence from Airbnb. Mark Sci. (2021) 40:23–47. doi: 10.1287/mksc.2020.1227, 19642375

[67] Young A. Channeling fisher: randomization tests and the statistical insignificance of seemingly significant experimental results. Q J Econ. (2019) 134:557–98. doi: 10.1093/qje/qjy029

[68] Conley TG Hansen CB Rossi PE. Plausibly exogenous. Rev Econ Stat. (2012) 94:260–72. doi: 10.1162/REST_a_00139

[69] Guo S. The legacy effect of unexploded bombs on educational attainment in Laos. J Dev Econ. (2020) 147:102527. doi: 10.1016/j.jdeveco.2020.102527

[70] Cinelli C Hazlett C. An omitted variable bias framework for sensitivity analysis of instrumental variables. Biometrika. (2025) 112:asaf004. doi: 10.1093/biomet/asaf004

[71] Hidalgo A. Your room is ready: tourism and urban revival. Reg Sci Urban Econ. (2024) 109:104059. doi: 10.1016/j.regsciurbeco.2024.104059

[72] Cinelli C Hazlett C. Making sense of sensitivity: extending omitted variable bias. J R Stat Soc Ser B Stat Methodol. (2020) 82:39–67. doi: 10.1111/rssb.12348

[73] Cornelissen T Dustmann C Raute A Schönberg U. From LATE to MTE: alternative methods for the evaluation of policy interventions. Labour Econ. (2016) 41:47–60. doi: 10.1016/j.labeco.2016.06.004

[74] Andresen ME. Exploring marginal treatment effects: flexible estimation using Stata. Stata J. (2018) 18:118–58. doi: 10.1177/1536867X1801800108

[75] Abadie A Athey S Imbens GW Wooldridge JM. When should you adjust standard errors for clustering? Q J Econ. (2023) 138:1–35. doi: 10.1093/qje/qjac038

[76] Jin T Liu H. The heterogeneous effect of post-compulsory education on subjective well-being: evidence based on marginal treatment effect. Appl Res Qual Life. (2023) 18:2851–76. doi: 10.1007/s11482-023-10210-y

[77] Olasehinde TS Jin Y Qiao F Mao S. Marginal returns on Chinese agricultural technology transfer in Nigeria: who benefits more? China Econ Rev. (2023) 78:101935. doi: 10.1016/j.chieco.2023.101935

[78] Sun Y Li M Pan Z Zhou X. Who benefits from compulsory education? Evidence from the average and heterogeneous effects on migrant welfare in China. China Econ Rev. (2026) 95:102559. doi: 10.1016/j.chieco.2025.102559

[79] Menassa M Stronks K Khatami F Roa Díaz ZM Pano Espinola O Gamba M . Concepts and definitions of healthy ageing: a systematic review and synthesis of theoretical models. eClinical Med. (2023) 56:101821. doi: 10.1016/j.eclinm.2022.101821, 36684393 PMC9852292

