# Supplementary material for: The impact of home- and community-based services on life satisfaction among older adults: evidence from marginal treatment effect framework
Source: Front Public Health. 2026 Jul 6;14:1852355. doi: 10.3389/fpubh.2026.1852355 (PMC13381838; doi:10.3389/fpubh.2026.1852355)
Supplement: Supplementary file 1 [file Data_Sheet_1.PDF]

Supplementary Material

1 Supplementary Figures and Tables

1.1 Supplementary Figures

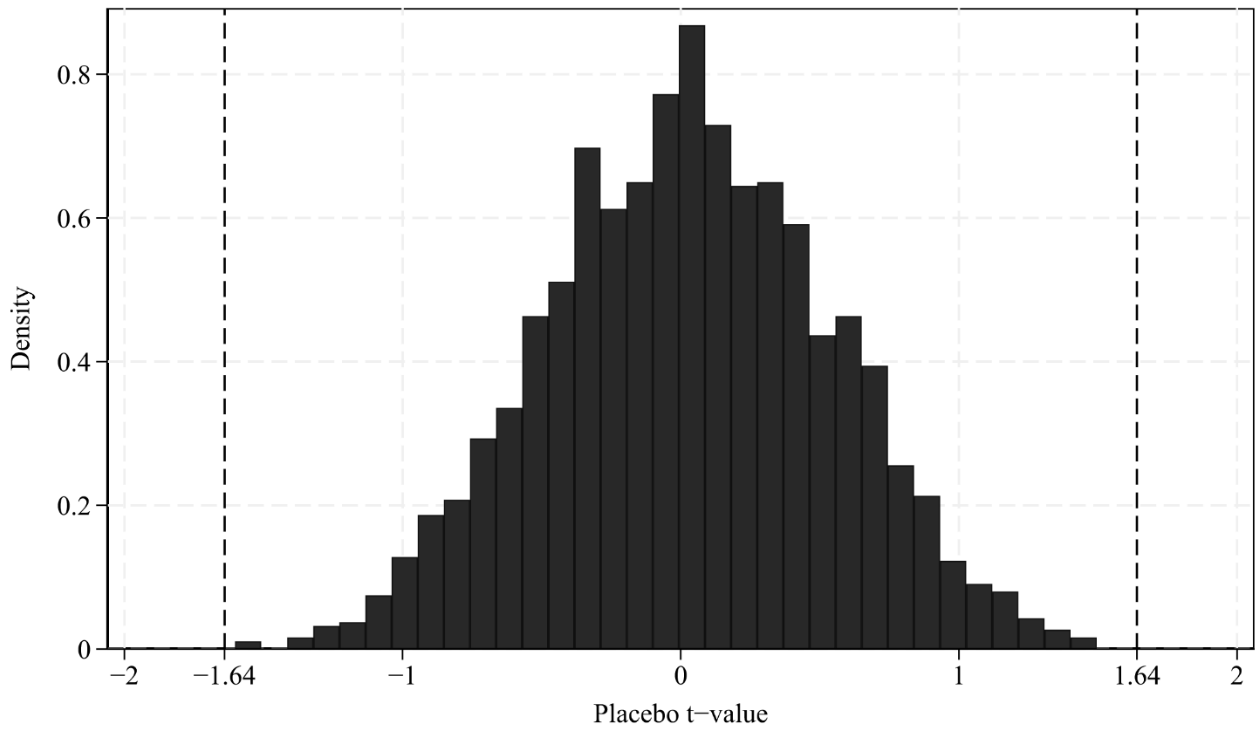

Supplementary Figure 1. Placebo test

Note: The figure shows the distribution of 2000 placebo t-values. The two vertical dashed lines indicate the 10% significance threshold for placebo t-values, i.e., ±1.64.

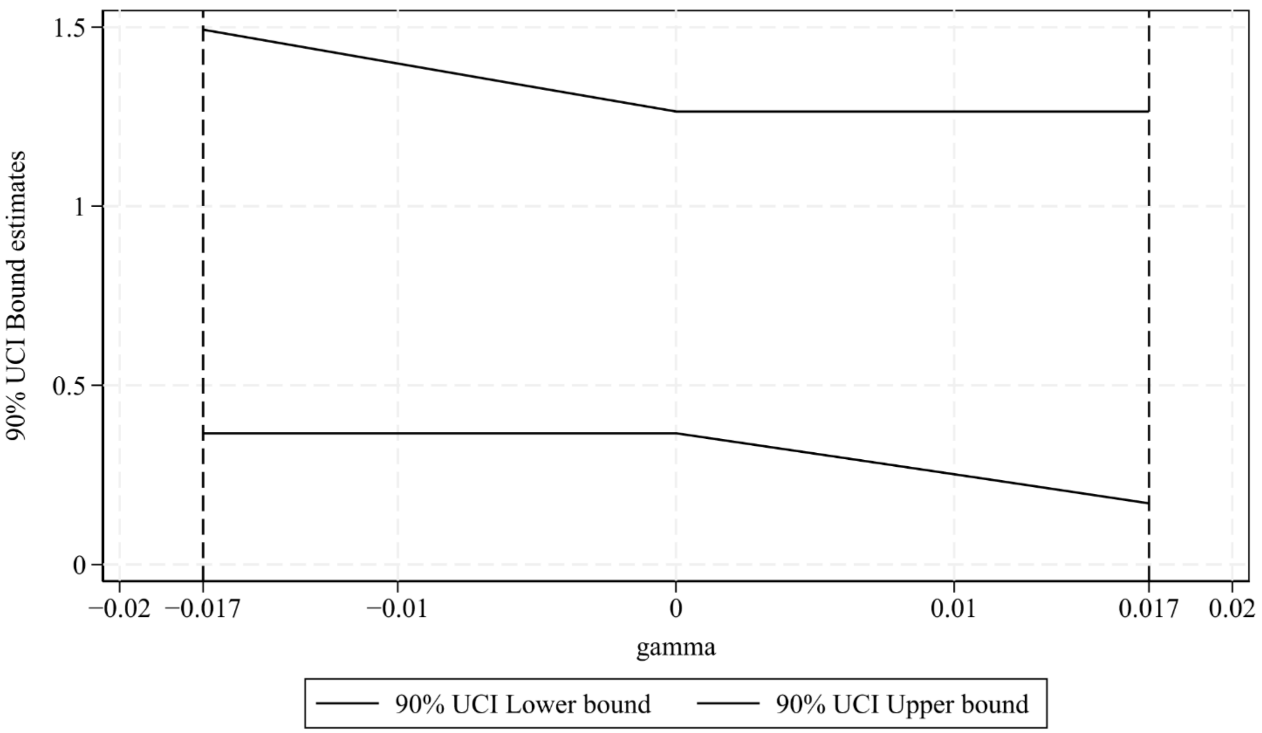

Supplementary Figure 2. Sensitivity analysis

Note: This figure plots the 90% union of confidence intervals (UCI) for the 2SLS estimates under a relaxed assumption on the IV's exclusion restriction. The two vertical dashed lines indicate the range of gamma used to compute the 90% UCI bound estimates, i.e., $\pm 0.25\beta_{RF}$, where β_{RF} denotes the coefficient of community service facilities in the reduced-form regression. Given that $\beta_{RF} = 0.066$, the range of gamma is [-0.017, 0.017].

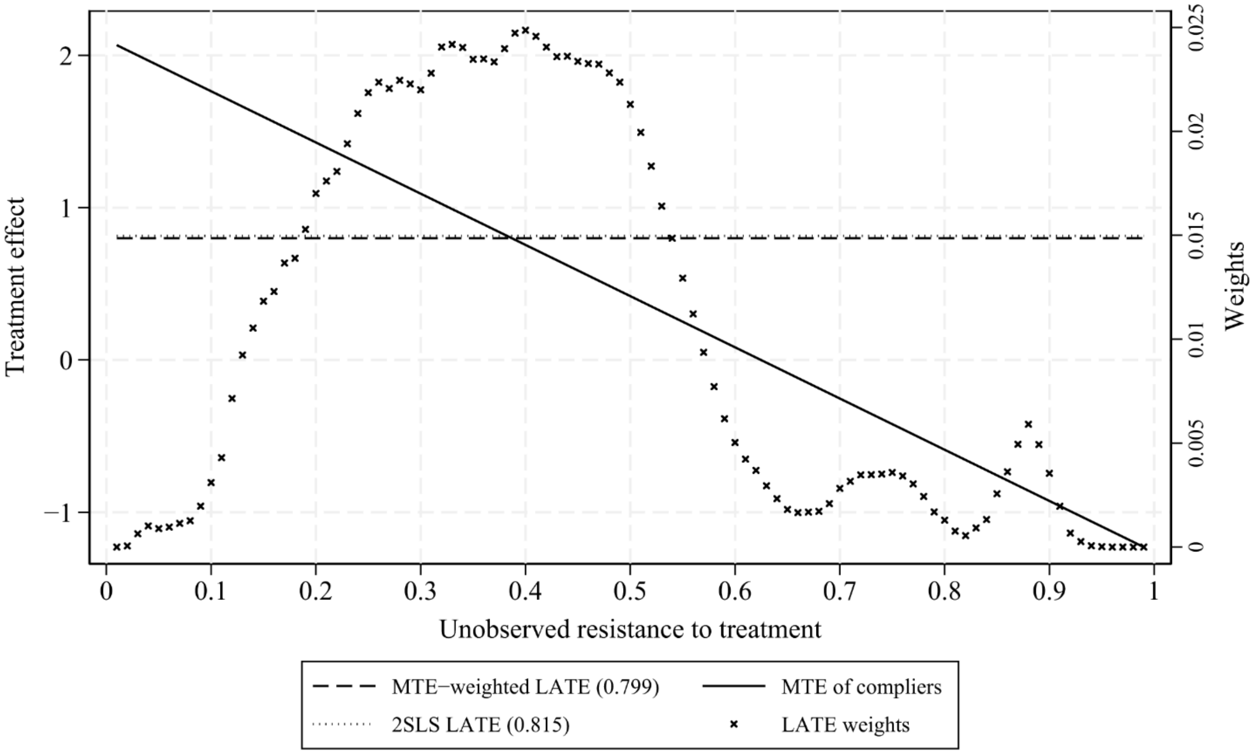

Supplementary Figure 3. LATE weights and estimates

Note: The figure plots the MTE estimates for compliers and the weights used to construct the LATE, and also reports LATE estimates obtained using the MTE-weighted approach and the 2SLS specification. The solid line represents MTE estimates for compliers, the dashed line represents the LATE estimated using the MTE-weighted method, and the dotted line represents the LATE estimated using the 2SLS specification. The left y-axis reports the values of these treatment effects. The cross markers represent the weights used to compute the MTE-weighted LATE, and the right y-axis reports the values of these weights.

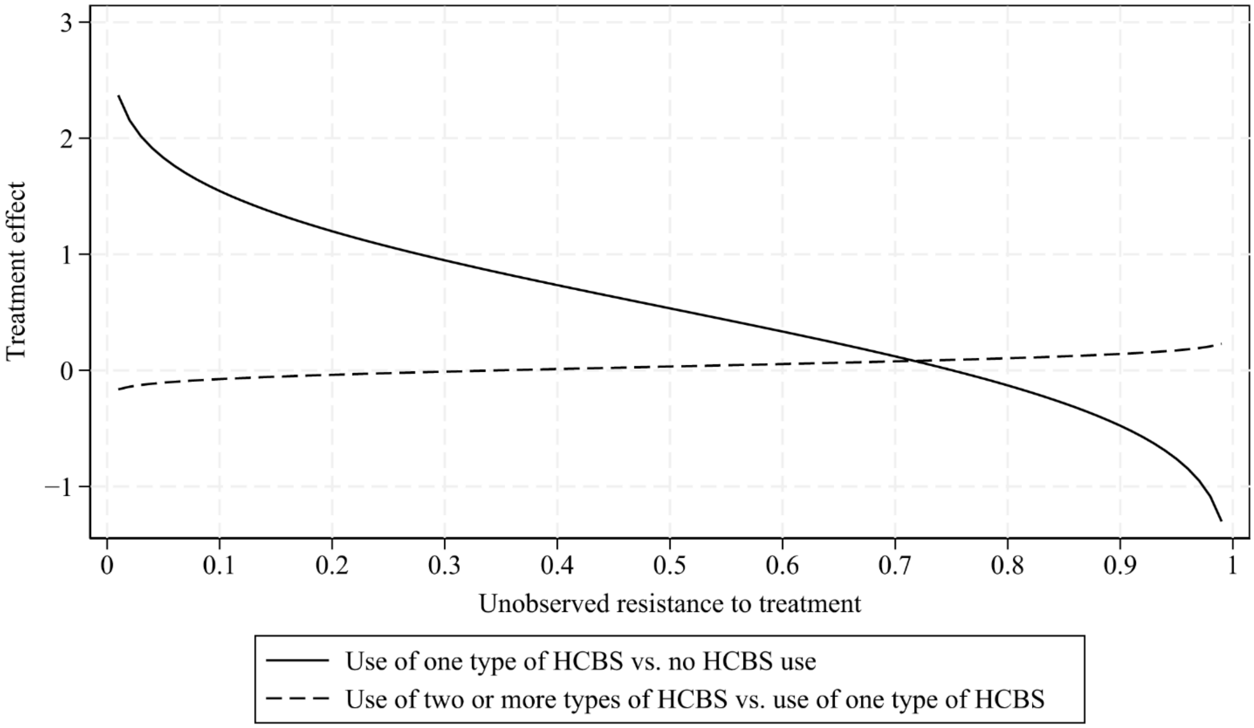

Supplementary Figure 4. Multivalued treatment MTE curves

Note: The figure presents MTE curves for two transitions in treatment status. Specifically, the solid line plots the MTE curve for the transition from no HCBS use to the use of one type of HCBS, while the dashed line plots the MTE curve for the transition from the use of one type of HCBS to the use of two or more types of HCBS.

1.2 Supplementary Tables

Supplementary Table 1. Variance-weighted Kolmogorov-Smirnov tests

	Trimming constants ξ		
	0.07	0.3	1
	(1)	(2)	(3)
p-values	0.33	0.25	0.22

Note: p-values from variance-weighted Kolmogorov-Smirnov tests under different trimming constants ξ (including 0.07, 0.3, and 1) are reported. Statistically significant p-values indicate that the null hypothesis that the IV is valid cannot be rejected.

Supplementary Table 2. Intersection bounds approach

	Significance levels		
	10%	5%	1%
	(1)	(2)	(3)
Intersection bounds	[-0.006, inf)	[-0.008, inf)	[-0.011, inf)

Note: Intersection bounds estimates at different significance levels (10%, 5%, and 1%) are reported. A lower intersection bound below zero indicates that the null hypothesis that the IV is valid cannot be rejected.

Supplementary Table 3. Robustness checks

	ATE	ATT	ATUT	Observable heterogeneity	Essential heterogeneity
**Panel A. Addressing attrition bias**					
IPAW	0.637**	1.417***	-0.003	<0.01	<0.01
	(0.246)	(0.313)	(0.364)		
Upper bound	0.536**	1.439***	-0.253	<0.01	<0.01
	(0.259)	(0.323)	(0.392)		
Lower bound	0.593**	1.386***	-0.099	<0.01	<0.01
	(0.263)	(0.317)	(0.404)		
**Panel B. Alternative model specifications**					
Logit	0.537**	1.401***	-0.218	<0.01	<0.01
	(0.251)	(0.308)	(0.382)		
LPM	0.503*	1.388***	-0.272	<0.01	<0.01
	(0.274)	(0.337)	(0.417)		
Cubic	0.617**	1.744***	-0.368	<0.01	<0.01
	(0.284)	(0.335)	(0.442)		
Normal	0.421*	1.078***	-0.152	<0.01	<0.01
	(0.240)	(0.325)	(0.341)		
Semiparametric	0.526*	1.339***	-0.204	<0.01	<0.01
	(0.275)	(0.424)	(0.341)		
**Panel C. MTE estimates for different categories of HCBS utilization**					
Medical service utilization	0.522*	1.497***	-0.246	<0.01	<0.01
	(0.290)	(0.326)	(0.447)		
Eldercare service utilization	-1.446	2.029*	-2.095	<0.01	<0.01
	(1.205)	(1.126)	(1.303)		
**Panel D. Alternative instrumental variables**					
Classifying community service facilities into two precise categories	0.382*	1.371***	-0.482	<0.01	<0.01
	(0.212)	(0.257)	(0.325)		
**Panel E. Changing the level of fixed effects**					
Controlling for community fixed effects	1.248***	1.828***	0.755	<0.01	0.09
	(0.338)	(0.382)	(0.530)		
**Panel F. Using clustered standard errors**					
Clustering standard errors at the community level	0.536*	1.414***	-0.232	<0.01	<0.01
	(0.282)	(0.394)	(0.401)		

Note: * $p < 0.1$, ** $p < 0.05$, *** $p < 0.01$. Robust standard errors in parentheses.

Supplementary Table 4. Multivalued treatment MTE estimates

Treatment status transition	ATE<br>(1)	Test for the slope of MTE curves<br>(2)
Use of one type of HCBS vs. no HCBS use	0.535***	-0.789***
	(0.182)	(0.270)
Use of two or more types of HCBS vs. use of one type of HCBS	0.033	0.085
	(0.244)	(0.242)

Note: * $p < 0.1$, ** $p < 0.05$, *** $p < 0.01$. Robust standard errors in parentheses.
